# TRIM21 Exacerbates Ischemic Brain Injury by Promoting Astrocyte-Mediated Neuroinflammation via K63-Linked Ubiquitination of MDA5

**DOI:** 10.34133/research.1200

**Published:** 2026-03-17

**Authors:** Yirui Kuang, Huaping Huang, Kaibo Yu, Tianchi Tang, Yonghe Zheng, Xian Yu, Linfeng Fan, Hang Zhou, Yin Li, Yinghan Guo, Yi Zhang, Fengqi Zhou, Jianru Li, Chi Gu, Junyou Wang, Liya Lin, Guannan Guan, Feng Yan, Gao Chen

**Affiliations:** ^1^ Department of Neurosurgery, Second Affiliated Hospital, School of Medicine, Zhejiang University, Hangzhou, China.; ^2^ Zhejiang Key Laboratory of Research and Transformation for Major Neurosurgical Diseases, Hangzhou, China.; ^3^ State Key Laboratory of Transvascular Implantation Devices, Hangzhou, China.; ^4^ Department of Radiology, Second Affiliated Hospital, School of Medicine, Zhejiang University, Hangzhou, China.; ^5^ Center for Basic and Translational Research, Second Affiliated Hospital, School of Medicine, Zhejiang University, Hangzhou, China.

## Abstract

The pathophysiology of ischemic stroke is critically mediated by detrimental neuroinflammation. Tripartite motif-containing 21 (TRIM21) serves as an E3 ubiquitin ligase that regulates important biological functions. Nonetheless, the specific role of TRIM21 in neuroinflammation and ischemic brain injury remains unclear. In this study, we found that TRIM21 expression was up-regulated in astrocytes within the peri-infarct regions of mice subjected to transient middle cerebral artery occlusion (tMCAO) and in an in vitro oxygen–glucose deprivation and reoxygenation (OGD/R) model. TRIM21 deficiency alleviated cerebral ischemia/reperfusion (I/R) injury by attenuating the inflammatory responses and oxidative stress. Mechanistically, our findings demonstrated that TRIM21 interacts with MDA5 and sequentially promotes the K63-linked ubiquitination and stabilization of MDA5, ultimately activating the nuclear factor κB (NF-κB) pathway in astrocytes. Moreover, MDA5 overexpression effectively reversed protective effects of TRIM21 deficiency after cerebral ischemia. Consistently, brain-targeted TRIM21 silencing with nanoparticle delivery considerably ameliorated cerebral I/R injury. Collectively, our findings identify TRIM21 as a novel astrocyte-specific mediator of neuroinflammation in cerebral ischemic injury and highlight its potential as a therapeutic target for ischemic stroke.

## Introduction

Ischemic stroke is a severe cerebrovascular disease associated with high morbidity, disability, and mortality rates, imposing a large health and economic burden worldwide [1]. Therapeutic progress in ischemic stroke, notably through thrombolysis and thrombectomy, has yielded marked improvements in patient outcomes [2]. Despite the success of recanalization therapies, many patients still experience unfavorable prognoses, often presenting with hemorrhagic transformation, reperfusion injury, or inadequate microcirculatory reperfusion. Rather than being isolated complications, these adverse outcomes are notable clinical-pathological manifestations of ischemic brain injury, which arises from multiple pathological processes. Among these, ischemia/reperfusion (I/R) injury constitutes the predominant driver that amplifies tissue damage and dysfunction [3]. Thus, there is an urgent need to elucidate the pathophysiological mechanisms that underlie ischemic brain damage and to investigate novel targets for ischemic stroke treatment.

The pathophysiology of cerebral ischemic stroke is complex and involves several mechanisms, such as calcium dysregulation, excitotoxicity, oxidative stress, mitochondrial dysfunction, and neuroinflammation. Notably, neuroinflammation represents a major and perpetuating element within the ischemic cascade following cerebral ischemia, playing a critical role in driving secondary damage for prolonged periods after the initial ischemic event [4]. The resident innate immune cells of the central nervous system (CNS), particularly microglia and astrocytes, mediate this sterile inflammatory response. As the most abundant type of glial cells, astrocytes are essential for maintaining and regulating CNS homeostasis under normal physiological conditions [5]. Astrocytes exhibit activation and abnormal morphology with reactive astrogliosis under pathological conditions. While astrocytes endeavor to protect the brain from damage by the secretion of anti-inflammatory mediators, the sustained activation and abnormal expression of inflammatory factors, along with the release of potentially neurotoxic mediators, contribute to the progression of ischemic stroke [6]. Growing evidence suggests that astrocyte-driven inflammation following ischemia exacerbates brain injury [7,8]. In their reactive state, astrocytes can polarize toward a deleterious neuroinflammatory phenotype, characterized by the sustained release of cytokines, chemokines, and other tissue damage-associated molecules that exacerbate neuronal injury and brain edema, intensify oxidative stress, and aggravate blood–brain barrier (BBB) permeability [9]. As a result, understanding the molecular mechanisms that govern astrocyte-driven inflammatory pathways is vital for identifying novel neuroprotective interventions for ischemic stroke.

TRIM (tripartite motif) proteins, most of which process E3 ubiquitin ligase activities, are involved in regulating a series of biological processes and diseases, including apoptosis, innate immune responses, autophagy, and tumorigenesis [10]. Previous studies have uncovered the functions of TRIM8/9/16/25/45/47/72 in ischemic stroke [11–13]. Like other TRIM family proteins, TRIM21 is a RING-type E3 ubiquitin ligase that regulates diverse cellular processes, including redox homeostasis, transcription, apoptosis, autophagy, and inflammation [14]. Accumulating evidence suggests that TRIM21 is involved in the development of CNS diseases. In support of these, TRIM21 has been found to facilitate the removal of pathological tau aggregates in Alzheimer’s disease, demonstrating its capacity to influence disease-related proteostasis [15]. Following subarachnoid hemorrhage, TRIM21 disrupts the BBB by promoting the ubiquitination of astrocytic β1-integrin, which results in early brain injury [16]. Moreover, up-regulated TRIM21 promotes PKM2 nuclear translocation-driven glycolytic reprogramming and astrocyte activation, thereby exacerbating neuroinflammation in experimental autoimmune encephalomyelitis [17]. Notably, emerging studies implicate TRIM21 in astrocyte dysfunction via discrete ubiquitin-dependent pathways, compromising BBB integrity and enforcing proinflammatory activation, which potentially exacerbates CNS injury. However, the function of TRIM21 in ischemic stroke has not been studied.

Melanoma differentiation-associated protein 5 (MDA5) is a cytosolic pattern recognition receptor that detects viral double-stranded RNA and initiates innate immune responses [18]. Upon activation, MDA5 facilitates the assembly of signaling complexes that subsequently activate downstream interferon regulatory factors (IRFs) and nuclear factor κB (NF-κB) signaling pathways. This leads to the production of type I interferons and pro-inflammatory cytokines, amplifying inflammatory signaling cascades. The stability and activity of MDA5 are tightly regulated by ubiquitination, a critical posttranslational modification mediated by the dynamic interplay between E3 ligases and deubiquitinating enzymes (DUBs). Recently, RNF144B has been found to promote MDA5 degradation via autophagy by mediating K27/K33-linked polyubiquitination at lysine residues 23 and 43, thereby negatively regulating antiviral responses [19]. Conversely, the DUB USP8 stabilizes MDA5 by removing K6/K27-linked ubiquitin chains, potentiating type I interferon and cytokine production [20]. Nevertheless, the expression, activation, and pathological functions of MDA5 in cerebral ischemia remain poorly understood. Of particular interest is whether the E3 ubiquitin ligase TRIM21, a known modulator of inflammatory processes, regulates MDA5 stability and function via ubiquitination, thereby influencing neuroinflammatory responses after ischemic injury.

In this study, we aimed to identify the role of TRIM21 in ischemic stroke using cellular and animal models. Here, we ​demonstrated​ that TRIM21 ​exacerbated​ infarct volumes, ​disrupted​ BBB integrity, and ​impaired​ functional recovery. ​Mechanistically,​​ TRIM21 ​interacted with​ MDA5 to promote its K63-linked ubiquitination and stabilization, which enhanced astrocyte-mediated neuroinflammation ​via ​NF-κB signaling pathways. Furthermore, we explored the potential of brain-targeted TRIM21 silencing using nanoparticle (NP) delivery as a therapeutic strategy for stroke, considering its future clinical applications. Therefore, TRIM21 may serve as a potential target for the treatment of ischemic stroke.

## Results

### TRIM21 deficiency attenuates ischemic brain damage and improves neurological recovery after tMCAO

**Fig. 1.**
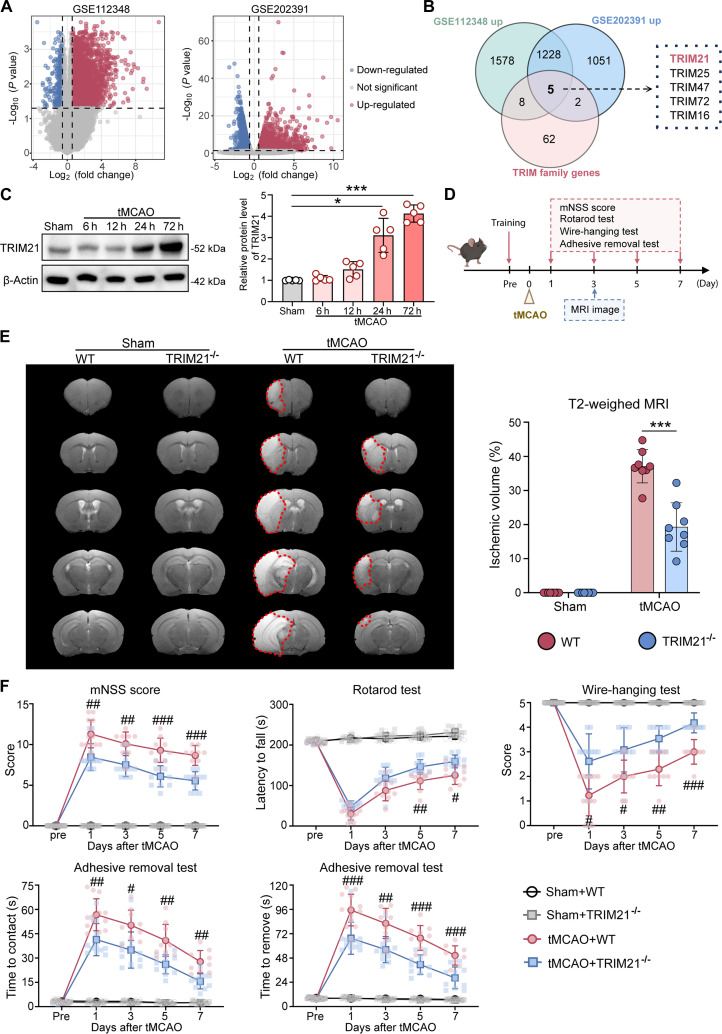
TRIM21 deficiency reduces brain ischemic volume and improves neurological recovery after tMCAO.​ (A) Volcano plots of differentially expressed genes from GSE112348 and GSE202391 datasets. (B) A Venn diagram highlighted 5 overlapping TRIM family genes, including TRIM21. (C) Western blot images and quantification of TRIM21 protein expression in ischemic brain tissue from WT mice at 6, 12, 24, and 72 h post-tMCAO (*n* = 5, Welch’s ANOVA followed by Dunnett’s T3 post hoc test). (D) Schematic diagram of experimental design for (E) and (F). (E) Representative T2-weighted MRI images and quantification of infarct volume in WT and TRIM21^−/−^ mice on day 3 post-tMCAO (*n* = 8, unpaired *t* test). (F) Behavioral tests, including mNSS score, rotarod test, wire-hanging test, and adhesive removal test, were assessed before the surgery and on days 1, 3, 5, and 7 post-tMCAO (*n* = 9 to 15, 2-way ANOVA followed by Tukey’s post hoc test). All data are expressed as means ± SD. *P < 0.05 and ***P < 0.001 versus indicated groups; ^#^*P* < 0.05, ^##^*P* < 0.01, and ^###^*P* < 0.001 versus tMCAO + TRIM21^−/−^ group.

To investigate the involvement of TRIM family members in cerebral I/R injury, we initiated our study by examining transcriptomic data from Gene Expression Omnibus (GEO) datasets (GSE112348, cortex; GSE202391, hippocampus) of mice subjected to transient middle cerebral artery occlusion (tMCAO) or sham operations. Differential gene expression analysis identified up-regulated genes (Fig. 1A), and their intersection with the TRIM family gene set revealed 5 consensus genes: TRIM21, TRIM25, TRIM47, TRIM72, and TRIM16 (Fig. 1B). Notably, TRIM21 had not been previously reported in the context of cerebral I/R injury, prompting us to focus on it for further investigation. Western blot analysis further demonstrated a time-dependent increase in TRIM21 protein levels following tMCAO, with peak levels observed at 72 h (Fig. 1C). To elucidate the role of TRIM21 in I/R injury, we generated TRIM21 knockout (KO) (TRIM21^−/−^) mice (Fig. S1). The experimental procedure was conducted as outlined for Fig. 1D. Both wild-type (WT) and TRIM21^−/−^ mice underwent tMCAO, and subsequent assessments of cerebral infarct volume and neurological functional recovery were performed. T2-weighted magnetic resonance imaging (MRI) conducted on day 3 post-tMCAO revealed significantly reduced infarct volumes in the ischemic hemispheres of TRIM21^−/−^ mice compared to WT mice (Fig. 1E). Furthermore, the KO of TRIM21 significantly mitigated functional impairments, as evidenced by a substantial reduction in scores on the modified neurological severity score (mNSS) test, prolonged rotarod endurance, improved performance in the wire-hanging test, and reduced contact/removal time in the adhesive removal test compared to WT mice at several time points (1, 3, 5, and 7 d) after tMCAO (Fig. 1F). Given the sex-associated differences in ischemic stroke [21], similar outcomes were observed in female mice, where TRIM21^−/−^ mice demonstrated reduced infarct sizes and enhanced neurological scores relative to WT mice (Fig. S2). Collectively, these findings indicate that TRIM21 deficiency reduces brain damage and promotes neurological recovery after I/R.

### TRIM21 deficiency alleviates BBB damage and neuronal apoptosis after tMCAO

**Fig. 2.**
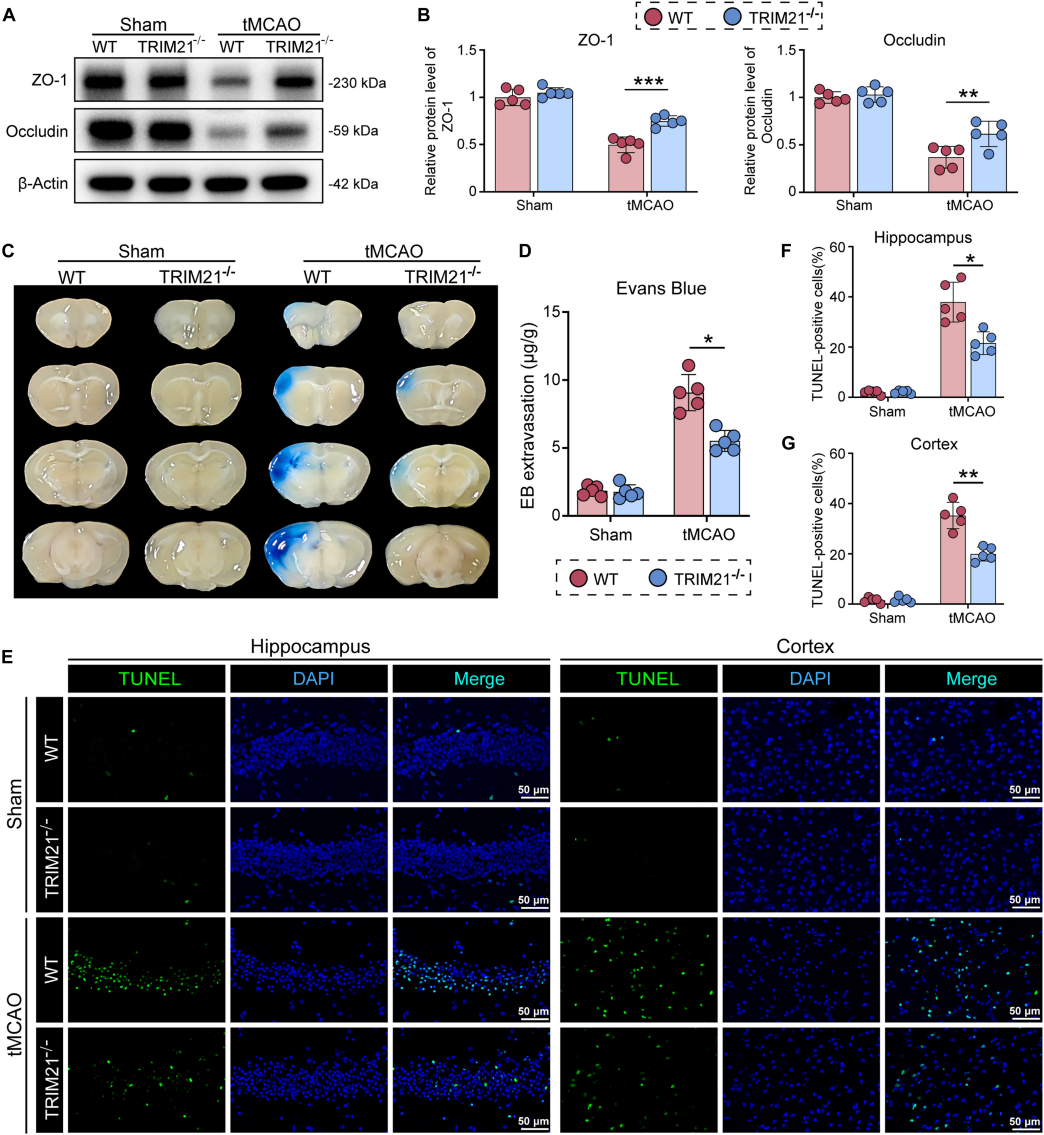
TRIM21 deficiency alleviates BBB damage and neuronal injury after tMCAO.​ (A and B) Western blot images and quantification of ZO-1 and Occludin protein expression in ischemic brain tissue of WT and TRIM21^−/−^ mice at 3 d post-tMCAO (*n* = 5, one-way ANOVA followed by Tukey’s post hoc test). (C and D) Representative images and quantitative analysis of the Evans Blue extravasation in the brain tissue of WT and TRIM21^−/−^ mice after tMCAO (*n* = 5, Welch’s ANOVA followed by Dunnett’s T3 post hoc test). (E) TUNEL staining demonstrating cell death in the peri-infarct hippocampus and cortex of WT and TRIM21^−/−^ mice. Scale bar, 50 μm. (F and G) Quantitative analysis of TUNEL-positive cells in the hippocampus (F) and cortex (G) (*n* = 5, Welch’s ANOVA followed by Dunnett’s T3 post hoc test). All data are expressed as means ± SD. *P < 0.05, **P < 0.01, and ***P < 0.001 versus indicated groups.

To address the limitations of T2-weighted MRI in distinguishing cerebral infarction from vasogenic edema during the subacute phase [22,23], we conducted an in-depth investigation into the changes in BBB permeability following I/R. The expression of tight junction proteins (ZO-1 and Occludin) was markedly reduced in the ischemic hemispheres of WT mice. Notably, TRIM21 KO significantly ameliorated the disruption of ZO-1 and Occludin at 3 d after tMCAO (Fig. 2A and B). This observation was corroborated by the Evans Blue (EB) permeability assay, which demonstrated a significant reduction in extravasation of the EB dye in the ischemic hemispheres of TRIM21^−/−^ mice (Fig. 2C and D). Additionally, terminal deoxynucleotidyl transferase–mediated deoxyuridine triphosphate nick end labeling (TUNEL) staining revealed a substantial decrease in TUNEL-positive cells within the cortex and hippocampus of TRIM21^−/−^ mice compared to WT mice at the same post-tMCAO time point (Fig. 2E to G). Taken together, these findings suggest that the ablation of TRIM21 enhances BBB integrity and reduces cellular apoptosis.

### TRIM21 is an astrocyte-specific regulator after ischemic stroke

**Fig. 3.**
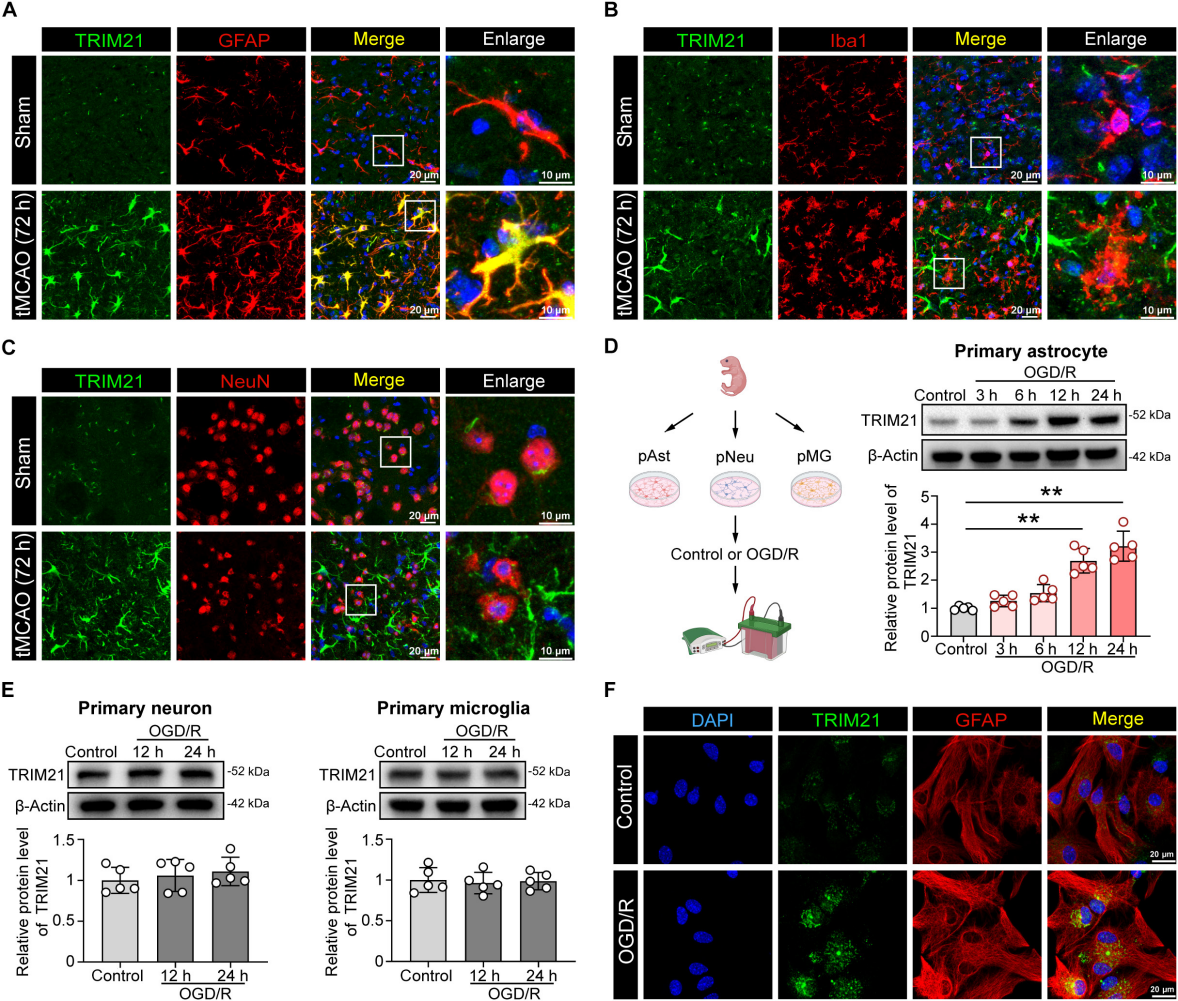
TRIM21 expression is up-regulated in astrocytes of the brain after ischemic stroke. (A to C) Immunofluorescence of TRIM21 in the peri-infarct region of the ischemic mice at 72 h post-tMCAO. Double immunofluorescence of TRIM21 (green) and GFAP (astrocyte marker, red) (A), Iba1 (microglia marker, red) (B), and NeuN (neuron marker, red) (C) was performed. Scale bars, 20 μm. (D) Western blot images and quantification of TRIM21 in primary astrocytes subjected to 6-h OGD, followed by 3, 6, 12, and 24 h of reoxygenation (*n* = 5, Welch’s ANOVA followed by Dunnett’s T3 post hoc test). (E) Western blot images and quantification of TRIM21 in primary neurons and microglia subjected to OGD (2 h for neurons and 3 h for microglia), followed by 12- and 24-h reoxygenation (*n* = 5, one-way ANOVA followed by Tukey’s post hoc test). (F) Representative immunofluorescence images of TRIM21 in primary astrocytes. Double immunofluorescence of TRIM21 (green) and GFAP (astrocyte marker, red) was performed. Scale bars, 20 μm. All data are expressed as means ± SD. **P < 0.01 versus indicated groups.

To determine which cell type shows increased TRIM21 expression in the ischemic hemisphere, we conducted co-immunofluorescence staining of TRIM21 with established markers: glial fibrillary acidic protein (GFAP) for astrocytes, Iba1 for microglia, and NeuN for neurons. Immunofluorescence staining indicated a significant up-regulation of TRIM21 expression in astrocytes located in the peri-infarct regions. In contrast, no such increase was observed in microglia or neurons at 72 h post-tMCAO (Fig. 3A to C and Fig. S3A). Corroborating these findings, single-cell transcriptomics revealed predominant up-regulation of Trim21 in astrocyte clusters (Fig. S3B to D). These observations were further supported using in vitro oxygen–glucose deprivation/reperfusion (OGD/R) models in primary astrocytes, microglia, and neurons. Western blot analysis showed a time-dependent induction of TRIM21 in astrocytes, with maximal expression observed at 24 h after OGD/R. In contrast, TRIM21 levels in neurons and microglia remained unchanged (Fig. 3D and E). Consistently, immunofluorescence analysis of OGD/R-treated astrocytes confirmed elevated TRIM21 expression (Fig. 3F). Taken together, these results reveal a predominant astrocytic up-regulation of TRIM21 following ischemia and may indicate their unique role in the pathophysiology of I/R injury.

### TRIM21 aggravates neuroinflammation and oxidative stress after cerebral I/R in vivo and astrocytic OGD/R in vitro

**Fig. 4.**
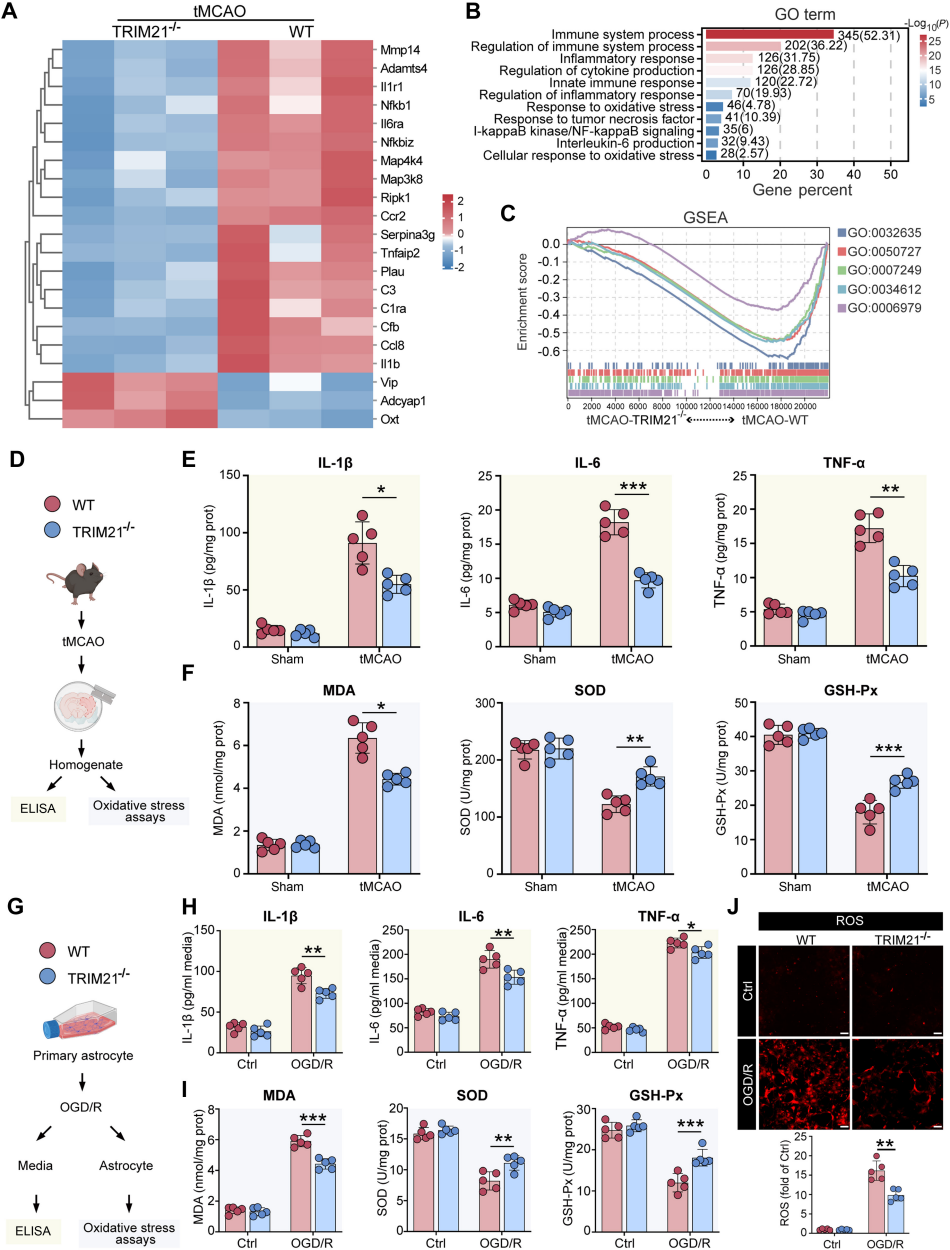
TRIM21 deficiency attenuates inflammation and oxidative stress following cerebral I/R injury in vivo and in vitro. (A to C) RNA-sequencing analysis in the ischemic hemispheres of WT and TRIM21^−/−^ mice at 72 h post-tMCAO (*n* = 3 mice per group). Heatmaps of gene expression (A), GO enrichment analysis (B), and Gene Set Enrichment Analysis (GSEA) (C) of tMCAO-TRIM21^−/−^ groups and tMCAO-WT groups. (D) Schematic diagram of experimental design for (E) and (F). (E and F) The ischemic hemispheres of WT and TRIM21^−/−^ mice at 72 h post-tMCAO were collected and homogenized to determine the levels of proinflammatory cytokines (IL-1β, IL-6, and TNF-α) (E) and oxidative stress (MDA, SOD, and GSH-Px) (F). *n* = 5. (G) Schematic diagram of experimental design for (H) and (I). (H) ELISA analysis of IL-1β, IL-6, and TNF-α levels in the culture medium of OGD/R-treated WT and TRIM21^−/−^ primary astrocytes. *n* = 5. (I) Effects of TRIM21 on MDA, SOD, and GSH-Px in OGD/R-treated primary astrocytes. *n* = 5. (J) Representative fluorescence staining images and quantitative analysis of ROS levels. Scale bars, 100 μm. *n* = 5. All data are expressed as means ± SD. In (E), IL-1β and TNF-α were analyzed by Welch’s ANOVA with Dunnett’s T3 test, while IL-6 was analyzed by one-way ANOVA with Tukey’s post hoc test. In (F), MDA was analyzed by Welch’s ANOVA with Dunnett’s T3 test, while SOD and GSH-Px were analyzed by one-way ANOVA with Tukey’s post hoc test. In (H) and (I), data were analyzed by one-way ANOVA with Tukey’s post hoc test. In (J), data were analyzed by Welch’s ANOVA with Dunnett’s T3 test. *P < 0.05, **P < 0.01, and ***P < 0.001 versus indicated groups.

To clarify the role of TRIM21 in cerebral I/R injury, we performed RNA sequencing analysis on WT and TRIM21^−/−^ mice 72 h post-tMCAO. The heatmap revealed a significant down-regulation of key genes associated with the inflammatory response, specifically Il1b, C3, Il6ra, Nfkb1, Ccl8, and Tnfaip2, in TRIM21^−/−^ mice compared to their WT counterparts (Fig. 4A). Functional enrichment analysis, including Gene Ontology (GO) and Gene Set Enrichment Analysis (GSEA), indicated a substantial involvement of differentially expressed genes in biological pathways related to inflammatory responses, including immune and inflammatory processes, regulation of cytokine production, IκB/NF-κB signaling, and responses to oxidative stress (Fig. 4B and C). Importantly, these findings suggest that the inflammatory and oxidative stress responses likely contribute to the phenotypic differences observed between WT and TRIM21^−/−^ mice following ischemic stroke. Thus, we focused on the specific effects of TRIM21 on post-ischemic inflammation and oxidative stress (Fig. 4D and G). Enzyme-linked immunosorbent assay (ELISA) conducted on homogenates from the ischemic hemispheres of tMCAO mice at 3 d post-tMCAO showed that TRIM21^−/−^ mice exhibited significantly lower protein levels of interleukin-1β (IL-1β), IL-6, and tumor necrosis factor-α (TNF-α) compared to WT mice (Fig. 4E). Reverse transcription quantitative real-time polymerase chain reaction (RT-qPCR) analysis further confirmed the decreased mRNA levels of these proinflammatory cytokines in TRIM21^−/−^ mice (Fig. S4A). Additionally, the absence of TRIM21 was associated with a reduction in oxidative stress, as evidenced by decreased levels of malondialdehyde (MDA) and enhanced activities of superoxide dismutase (SOD) and glutathione peroxidase (GSH-Px) in the ischemic hemispheres of tMCAO-TRIM21^−/−^ mice (Fig. 4F). Consistent with the in vivo findings, the secretion (Fig. 4H) and mRNA levels (Fig. S4B) of pro-inflammatory cytokines, including IL-1β, IL-6, and TNF-α, were dramatically increased after OGD/R in primary astrocytes, which were suppressed due to the loss of TRIM21 in vitro. Moreover, the knockdown of TRIM21 resulted in decreased reactive oxygen species (ROS) production and MDA levels while enhancing antioxidant enzyme activities (SOD and GSH-Px) in primary astrocytes after OGD/R (Fig. 4I and J). Collectively, these results indicate that TRIM21 exacerbates ischemic brain injury by amplifying neuroinflammatory and oxidative stress.

### TRIM21 interacts directly with MDA5

**Fig. 5.**
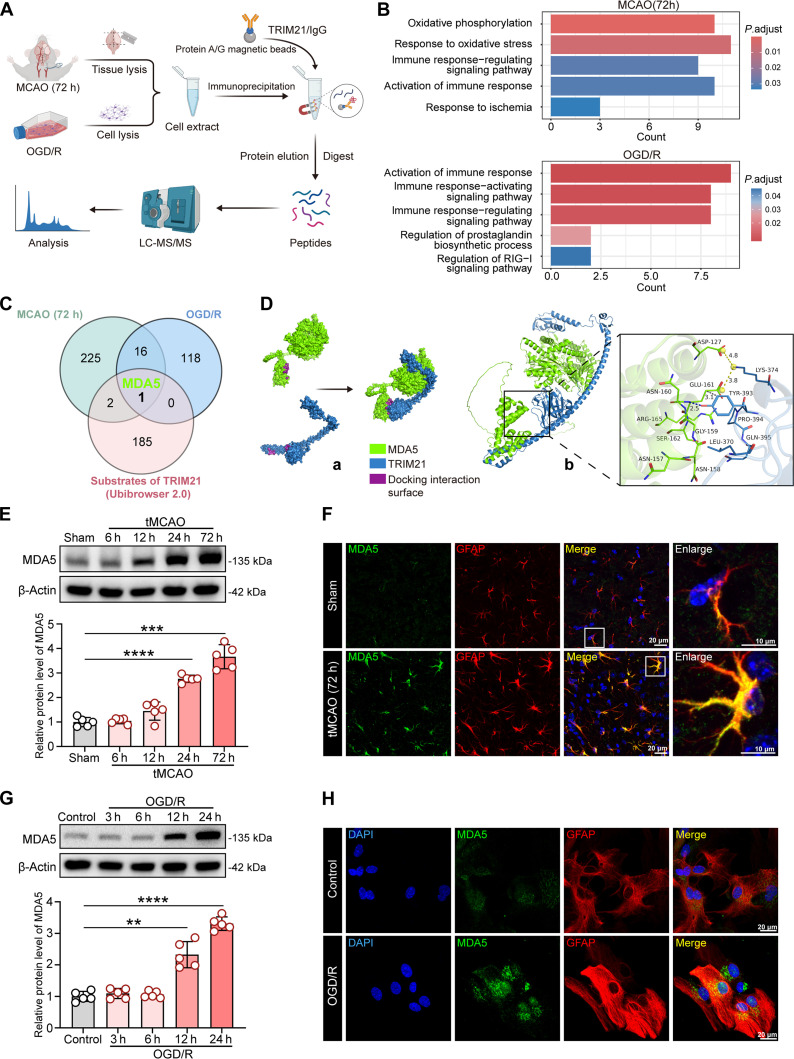
MDA5 is a potential interaction partner for TRIM21. (A) The schematic diagram showed that LC-MS/MS analysis was used to identify proteins interacting with TRIM21. (B) Gene Ontology (GO) enrichment analysis of potential interaction partners for TRIM21 in vivo and in vitro models. (C) The Venn diagram illustrated the proteins identified in both the LC-MS/MS databases and the Ubi-Browser dataset. (D) Protein–protein docking visualization (green for MDA5, blue for TRIM21). (a) Docking interaction surfaces of TRIM21 and MDA5 (indicated in purple). (b) Microscopic diagram showing the mechanism of TRIM21–MDA5 interaction. (E) Western blot images and quantification of MDA5 protein expression in ischemic brain tissue from WT mice at 6, 12, 24, and 72 h post-tMCAO (*n* = 5, Welch’s ANOVA followed by Dunnett’s T3 post hoc test). (F) Immunofluorescence of MDA5 in the peri-infarct region of the ischemic mice at 72 h post-tMCAO. Double immunofluorescence of MDA5 (green) and GFAP (astrocyte marker, red) was performed. Scale bars, 20 μm. (G) Western blot images and quantification of MDA5 in primary astrocytes subjected to 6-h OGD, followed by 3, 6, 12, and 24 h of reoxygenation (*n* = 5, Welch’s ANOVA followed by Dunnett’s T3 post hoc test). (H) Representative immunofluorescence image of MDA5 in primary astrocytes. Double immunofluorescence of MDA5 (green) and GFAP (astrocyte marker, red) was performed. Scale bars, 20 μm. All data are expressed as means ± SD. **P < 0.01, ***P < 0.001, and ****P < 0.0001 versus indicated groups.

To elucidate the molecular mechanism by which TRIM21 regulates inflammation and oxidative stress in cerebral I/R injury, we conducted co-immunoprecipitation (Co-IP) using a TRIM21-specific antibody [with immunoglobulin G (IgG) as a control] on ischemic hemisphere tissue collected 72 h post-tMCAO and on primary astrocytes subjected to OGD/R. Subsequently, immunoprecipitated complexes were subjected to liquid chromatography–tandem mass spectrometry (LC-MS/MS) analysis (Fig. 5A). In alignment with transcriptome sequencing data, GO analysis indicated that proteins interacting with TRIM21 were significantly enriched in biological processes associated with immune response modulation and oxidative stress (Fig. 5B). TRIM21 functions as an E3 ubiquitin ligase, typically involved in the ubiquitination of various substrates. Consequently, we integrated the Ubi-Browser 2.0 database with our LC-MS/MS analysis data to predict potential substrates of TRIM21. Notably, only MDA5 was identified as a potential substrate of TRIM21 (Fig. 5C). As a key cytosolic sensor in the RIG-I-like receptor (RLR) family, MDA5 is pivotal for activating immune responses, regulating inflammation, and mediating antiviral immunity. To date, its role in cerebral I/R injury remains unexplored. AlphaFold3 structural prediction revealed a plausible interaction interface between the P-SPRY domain of TRIM21 and the CARD domain of MDA5 (Fig. 5D). Thus, we conducted a detailed analysis of MDA5 expression and localization. A time-dependent increase in MDA5 protein levels was detected in ischemic hemispheres of WT mice after tMCAO, with a peak at 72 h. This was also observed in primary astrocytes subjected to OGD/R, with a peak at 24 h (Fig. 5E and G). Furthermore, immunofluorescence revealed specific colocalization of MDA5 with the astrocytic marker GFAP both in vivo and in vitro (Fig. 5F and H).

**Fig. 6.**
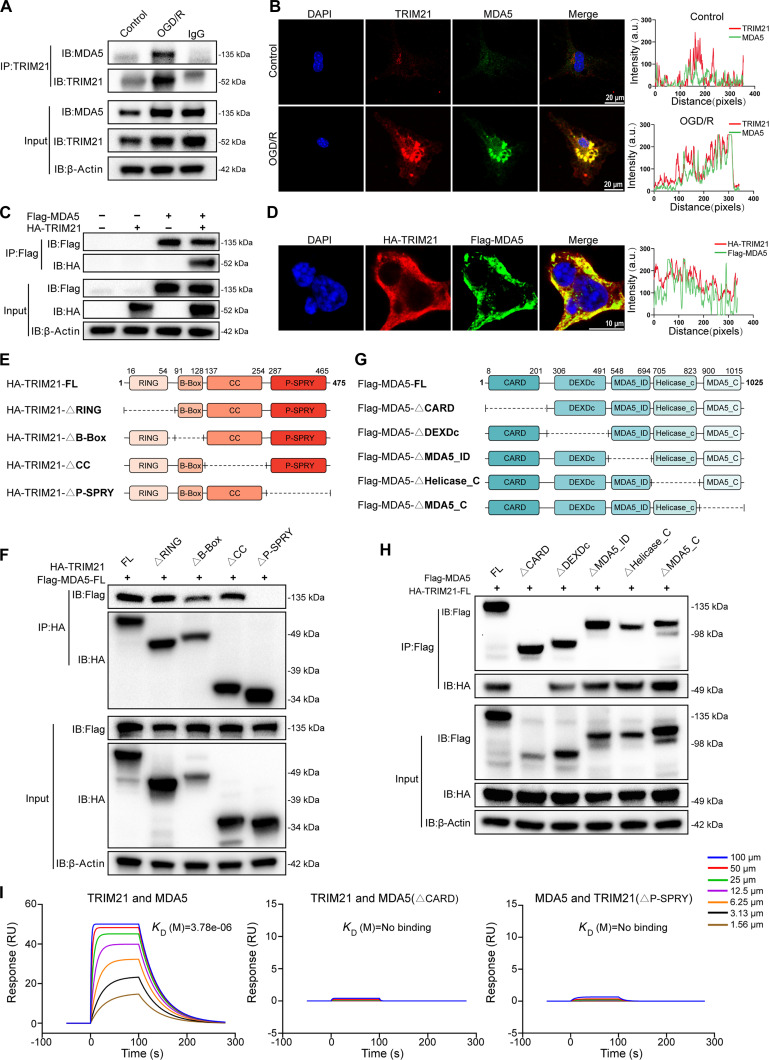
TRIM21 interacts with MDA5. (A) IP analysis of the endogenous interaction of TRIM21 with MDA5 in primary astrocytes. (B) Representative images of laser scanning confocal microscopy for TRIM21 (red) and MDA5 (green) in primary astrocytes subjected to OGD/R. The following 2 panels indicate the line profiling of TRIM21 with MDA5 under control and OGD/R stimulation conditions, respectively, and the intensity of each line was quantified by ImageJ software and drawn by GraphPad Prism 10.0. Scale bars, 20 μm. (C) IP analysis of the exogenous interaction of TRIM21 with MDA5 in HEK293T cells transfected with plasmids expressing HA-TRIM21 and Flag-MDA5. (D) Representative images of laser scanning confocal microscopy for HA (red) and Flag (green) in HEK293T cells. The following panel indicates the line profiling of exogenous TRIM21 with MDA5, and the intensity of each line was quantified by ImageJ software and drawn by GraphPad Prism 10.0. Scale bars, 10 μm. (E) Schematic diagram of TRIM21 and its truncation mutants. (F) HA-tagged TRIM21 or its mutants and Flag-MDA5 were individually transfected into HEK293T cells. The cell lysates were immunoprecipitated with an anti-HA antibody and then immunoblotted with the indicated antibody. (G) Schematic diagram of MDA5 and its truncation mutants. (H) Flag-tagged MDA5 or its mutants and HA-TRIM21 were individually transfected into HEK293T cells. The cell lysates were immunoprecipitated with an anti-Flag antibody and then immunoblotted with the indicated antibody. (I) Surface plasmon resonance (SPR) analysis for direct TRIM21–MDA5 interaction. Results are representative of 3 independent experiments.

Subsequently, we assessed the interaction between endogenous TRIM21 and MDA5 in primary astrocytes by Co-IP and found that it was robustly enhanced after OGD/R stimulation (Fig. 6A). This observation was backed by confocal microscopy, demonstrating the colocalization of TRIM21 and MDA5 in primary astrocytes (Fig. 6B). Additionally, hemagglutinin (HA)-TRIM21 and Flag-MDA5 plasmids were coexpressed in HEK293T cells, where exogenous Co-IP and confocal microscopy confirmed their interaction and colocalization (Fig. 6C and D). To identify the functional domains responsible for the TRIM21–MDA5 interaction, we performed Co-IP assays with a set of truncated mutants of both proteins (Fig. 6E and G). The results showed that TRIM21-△P-SPRY (the P-SPRY domain deletion mutant of TRIM21) was unable to interact with MDA5 (Fig. 6F). Similarly, MDA5-△CARD (the CARD domain deletion mutant of MDA5) lost the ability to interact with TRIM21 (Fig. 6H). Additionally, surface plasmon resonance (SPR) assays provided quantitative evidence of the direct interaction between TRIM21 and MDA5, which was abrogated by deletion of the relevant domains (Fig. 6I). Therefore, these data conclusively establish that TRIM21 interacts with MDA5 through specific binding between its P-SPRY domain and the CARD domain of MDA5.

### TRIM21 promotes K63-linked polyubiquitination of MDA5 to activate NF-κB signaling pathway

**Fig. 7.**
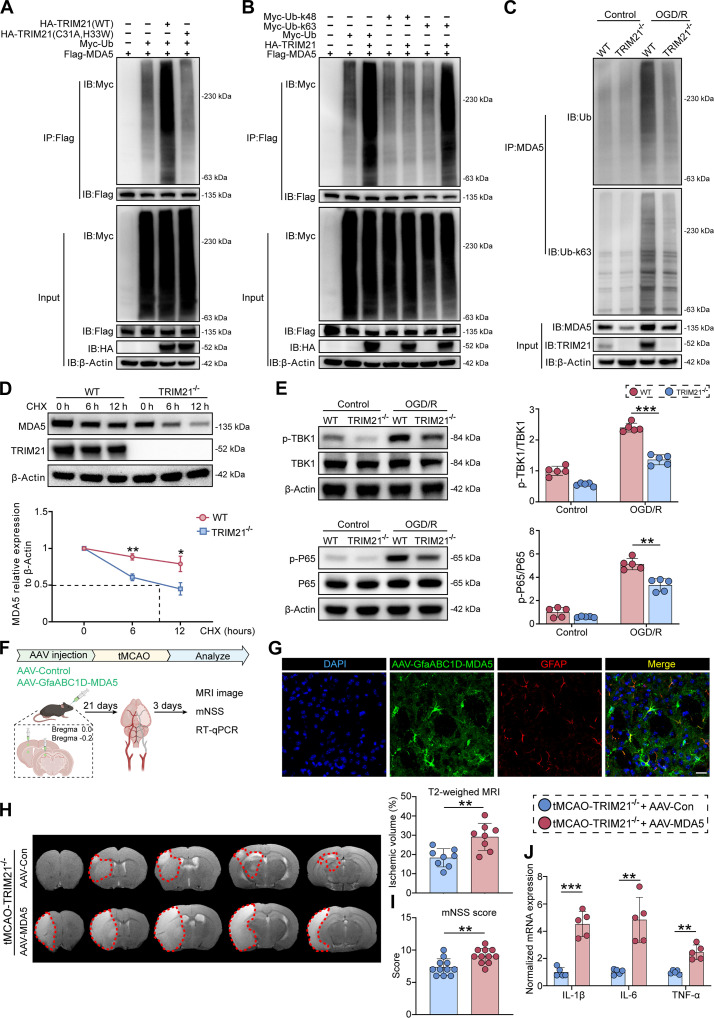
TRIM21 mediates K63-linked polyubiquitination of MDA5 to enhance NF-κB signaling and exacerbates ischemic brain injuries. (A) IP analysis of the ubiquitination of MDA5 in HEK293T cells transfected with plasmids expressing Flag-MDA5, Myc-ubiquitin (Myc-Ub), and HA-TRIM21 (WT or C31A, H33W). (B) HEK293T cells were transfected with plasmids expressing Myc-Ub (WT or K48, K63), HA-TRIM21, and Flag-MDA5. IP analysis of the polyubiquitination forms of MDA5 mediated by TRIM21. (C) Endogenous ubiquitination levels of MDA5 in WT and TRIM21^−/−^ primary astrocytes were examined by IP and Western blot. (D) Half-life of MDA5 was measured by the CHX assay in WT and TRIM21^−/−^ primary astrocytes (*n* = 3, 2-way ANOVA followed by Tukey’s post hoc test). (E) Western blot images and quantification of phosphorylation level of TBK1 and P65 in WT and TRIM21^−/−^ primary astrocytes after OGD/R (*n* = 5, Welch’s ANOVA followed by Dunnett’s T3 post hoc test). (F) Schematic diagram of experimental design for (G) to (J). (G) Representative immunofluorescence images of GFAP (red) and MDA5 (green) in AAV-GfaABC1D-MDA5-infected mice on day 21 after injection. Scale bars, 20 μm. (H) Representative T2-weighted MRI images and quantification of infarct volume in AAV-infected TRIM21^−/−^ mice on day 3 post-tMCAO (*n* = 8, unpaired *t* test). (I) The mNSS test was assessed in AAV-infected TRIM21^−/−^ mice on day 3 post-tMCAO (*n* = 11, unpaired *t* test). (J) RT-qPCR analysis of proinflammatory cytokine mRNA levels in ischemic hemispheres of AAV-infected TRIM21^−/−^ mice on day 3 post-tMCAO (*n* = 5, Welch’s *t* test). All data are expressed as means ± SD. *P < 0.05, **P < 0.01, and ***P < 0.001 versus indicated groups.

Based on the identification of E3 ubiquitin ligase TRIM21 as an MDA5-associated protein, we further investigated whether TRIM21 mediated the ubiquitination of MDA5. In HEK293T cells, cotransfection with Flag-tagged MDA5 and Myc-tagged ubiquitin, alongside either WT TRIM21 or TRIM21 RING domain mutants (C31A, H33W), demonstrated a significant increase in MDA5 ubiquitination when cotransfected with the WT TRIM21 expression plasmid. In contrast, the TRIM21 point mutation (C31A, H33W) failed to promote the polyubiquitination of MDA5, suggesting that the polyubiquitination of MDA5 depends on the E3 ligase activity of TRIM21 (Fig. 7A). Subsequently, we investigated which form of MDA5 polyubiquitination was mediated by TRIM21. As shown in Fig. 7B, TRIM21 predominantly promoted K63-linked, rather than K48-linked, polyubiquitination of MDA5. To validate the role of TRIM21 in MDA5 polyubiquitination under physiological conditions, we examined K63-linked polyubiquitination levels in WT and TRIM21^−/−^ primary astrocytes following OGD/R. Our results indicated a reduction in K63-linked polyubiquitination of MDA5 in TRIM21^−/−^ primary astrocytes (Fig. 7C). Notably, K63-linked polyubiquitination is a critical posttranslational modification that stabilizes proteins and facilitates signal transduction [24]. Previous studies have demonstrated that MDA5 can mobilize the TBK1 signaling cascade and amplify the NF-κB response [25]. In line with this, MDA5 knockdown inhibited OGD/R-induced TBK1/P65 phosphorylation in primary astrocytes (Fig. S5A). Consequently, we hypothesized that TRIM21-mediated K63-linked polyubiquitination could enhance MDA5 stability and promote NF-κB signaling. To test this hypothesis, we used cycloheximide (CHX) to inhibit de novo protein synthesis and subsequently monitored the degradation of MDA5 protein. Our findings revealed that TRIM21 deficiency markedly accelerated the degradation of MDA5 in primary astrocytes (Fig. 7D). We next assessed whether TRIM21-mediated MDA5 ubiquitination facilitated MDA5 activation. Semidenaturing detergent agarose gel electrophoresis (SDD-AGE) analysis revealed that OGD/R triggered MDA5 oligomerization and subsequent mitochondrial antiviral signaling protein (MAVS) aggregation in primary astrocytes, an effect reversed by TRIM21 deficiency (Fig. S5B). Additionally, TRIM21 deficiency reduced OGD/R-induced phosphorylation of TBK1 and P65 (Fig. 7E), suggesting impaired activation of the NF-κB signaling pathway.

To verify that MDA5 is the target through which TRIM21 could mediate ischemic stroke injury in vivo, we injected adeno-associated virus (AAV)-Control or AAV-MDA5 targeting astrocytes for MDA5 overexpression into the brains of TRIM21^−/−^ mice 3 weeks before tMCAO (Fig. 7F). Immunofluorescence results confirmed the selective expression of MDA5 in astrocytes (Fig. 7G). Furthermore, Western blot analysis of isolated astrocytes confirmed its effective overexpression (Fig. S6A). The overexpression of MDA5 in TRIM21^−/−^ mice exacerbated cerebral infarct volume and impaired neurological recovery (Fig. 7H and I). Furthermore, this was accompanied by a concomitant up-regulation of pro-inflammatory cytokines after tMCAO (Fig. 7J). Together, these findings suggest that TRIM21 exacerbates neuroinflammation by targeting MDA5, thereby aggravating tMCAO-induced brain injury.

### Engineered NP-mediated delivery of targeted TRIM21 silencing promotes neurological recovery in vivo

**Fig. 8.**
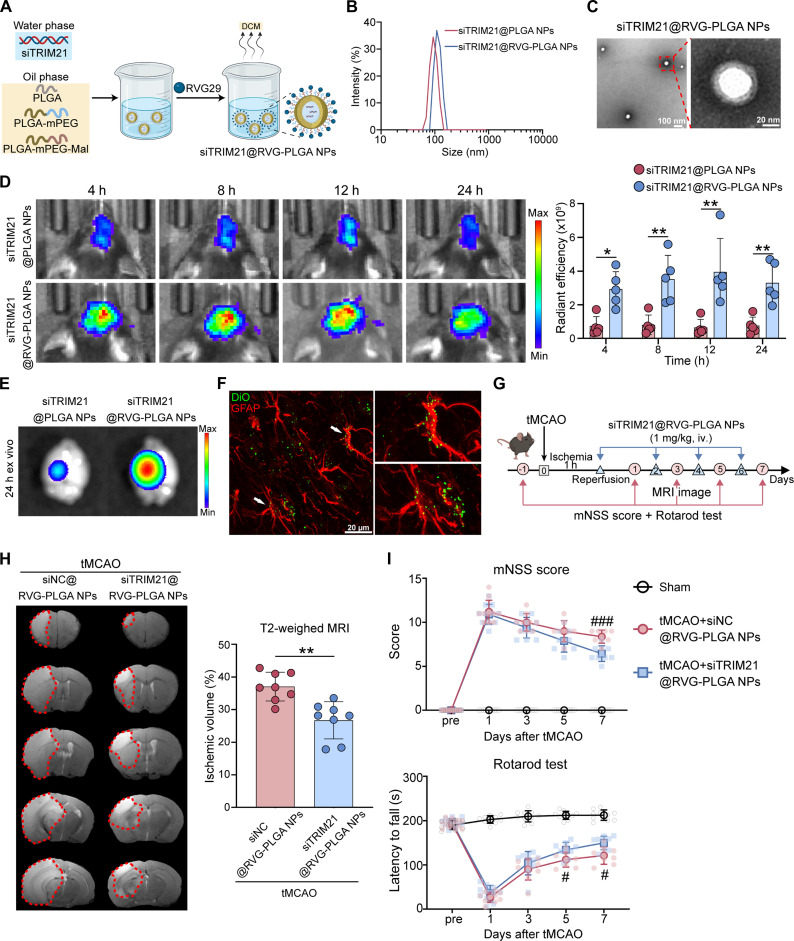
Engineered nanoparticle-mediated delivery of targeted TRIM21 silencing promotes neurological recovery after cerebral I/R. (A) Schematic illustration of the preparation of siTRIM21@RVG-PLGA NPs. (B) Evaluation of siTRIM21@PLGA NPs and siTRIM21@RVG-PLGA NPs detected by dynamic light scattering. (C) Transmission electron microscopy image of siTRIM21@RVG-PLGA NPs. Scale bars, 100 nm. (D) Pseudo-color images showed the DiR fluorescence of the in vivo brain 4, 8, 12, and 24 h after injection, and the scatterplot showed radiant efficiency (*n* = 5, 2-way ANOVA followed by Tukey’s post hoc test). (E) Pseudo-color images showed the DiR fluorescence of the ex vivo brain 24 h after injection. (F) Representative images of GFAP staining (red) in brain sections derived from mice treated with siTRIM21@RVG-PLGA NPs-DiO (green) at 24 h post-injection. (G) Schematic diagram of experimental design for (H) and (I). (H) Representative T2-weighted MRI images and quantification of infarct volume in the mice treated with siTRIM21@RVG-PLGA NPs or siNC@RVG-PLGA NPs on day 3 post-tMCAO (*n* = 8, unpaired *t* test). (I) Behavioral test results of the mNSS score and rotarod test from the mice treated with siTRIM21@RVG-PLGA NPs or siNC@RVG-PLGA NPs on days 1, 3, 5, and 7 post-tMCAO (*n* = 8 to 14, 2-way ANOVA followed by Tukey’s post hoc test). All data are expressed as means ± SD. *P < 0.05 and **P < 0.01 versus indicated groups; ^#^*P* < 0.05 and ^###^*P* < 0.001 versus tMCAO + siTRIM21@RVG-PLGA NPs group.

In the absence of available small-molecule inhibitors targeting TRIM21, we developed a biodegradable polymer NP platform for the systemic delivery of siTRIM21 in stroke therapy. It is crucial to recognize that the BBB poses a major challenge to the efficient penetration of NPs into the brain [26]. Thus, we engineered a NP system modified with RVG29 (a BBB-penetrating peptide) to enhance the delivery of siTRIM21 to the brain. Specifically, this system loading siTRIM21 was constructed by a combination of PLGA20K, PLGA20K-mPEG2K, and PLGA20K-mPEG5K-Mal, which were subsequently modified with the RVG29 peptide. The final product was RVG29-modified poly(lactic-co-glycolic acid) (PLGA) NPs loaded with siTRIM21, abbreviated as siTRIM21@RVG-PLGA NPs (Fig. 8A). The dynamic light scattering analysis revealed an average particle size of less than 200 nm, accompanied by a narrow size distribution (Fig. 8B). Transmission electron microscopy further confirmed that siTRIM21@RVG-PLGA NPs exhibited a relatively uniform spherical morphology (Fig. 8C). As depicted in Fig. 8D, 1,1′-dioctadecyl-3,3,3′,3′-tetramethylindotricarbocyanine iodide (DiR) fluorescence was prominently accumulated in specific regions of the brain as early as 4 h posttreatment with siTRIM21@RVG-PLGA NPs, and this accumulation persisted at elevated levels over time. Despite a significant uptake of NPs by the liver, ex vivo organ imaging analysis demonstrated that substantial amounts of NPs were still present in brain tissues (Fig. 8E and Fig. S6B). Additionally, immunofluorescence staining, which colabeled DiO-labeled siTRIM21@RVG-PLGA NPs with GFAP, indicated that siTRIM21@RVG-PLGA NPs were effectively absorbed by astrocytes (Fig. 8F and Fig. S6C). Critically, in vivo administration of siTRIM21@RVG-PLGA NPs led to a significant knockdown of TRIM21 protein levels specifically in astrocytes (Fig. S6D), demonstrating the efficacy of the delivery system.

Next, we investigated the effects of siTRIM21@RVG-PLGA NPs on neurological recovery after tMCAO in mice. The administration of siTRIM21@RVG-PLGA NPs or control NPs (siNC@RVG-PLGA NPs) took place at the time of reperfusion and continued on days 2, 4, and 6, with a dosage of 1 mg/kg delivered via tail vein injections [27]. We determined the cerebral infarct volume on day 3 post-tMCAO, with neurological function evaluated serially on postoperative days 1, 3, 5, and 7 (Fig. 8G). T2-weighted MRI results indicated a significant reduction in infarct volume in the group treated with siTRIM21@RVG-PLGA NPs (Fig. 8H). Furthermore, behavioral tests demonstrated enhanced neurological function, evidenced by decreased mNSS scores and improved rotarod test performance on days 5 and 7 (Fig. 8I). Thus, the aforementioned results suggest that siTRIM21@RVG-PLGA NPs are protective against I/R injury. A comprehensive evaluation of systemic toxicity following the injection of siTRIM21@RVG-PLGA NPs was conducted. There were no notable changes observed in routine blood indices, including red blood cells (RBC), white blood cells (WBC), platelets (PLT), and neutrophils (NEU), as well as in blood biochemical parameters such as aspartate aminotransferase (AST), alanine aminotransferase (ALT), blood urea nitrogen (BUN), and creatinine (CRE) on both day 1 and day 7 post-injection, when compared to control groups (Fig. S7A and B). Additionally, hematoxylin and eosin (H&E) staining of essential organs such as the brain, heart, liver, kidney, spleen, and lung revealed no signs of inflammation or tissue damage (Fig. S7C).

## Discussion

In our study, TRIM21 was identified as a newly recognized mediator of neuroinflammation and ischemic brain injury. We demonstrated that TRIM21 was up-regulated in astrocytes following cerebral ischemia and interacted with MDA5 to promote its K63-linked polyubiquitination and stabilization, thereby activating the NF-κB pathway and amplifying neuroinflammatory and oxidative stress responses. Genetic KO or NP-mediated silencing of TRIM21 significantly attenuated brain injury and improved functional recovery, highlighting its potential as a therapeutic target in ischemic stroke.

As a member of the TRIM protein family, TRIM21 is noted for its E3 ligase activity, which is attributed to its RING finger domain. The RING finger protein family participates in a variety of biological processes. Previous research has highlighted the crucial involvement of TRIM family members in ischemic stroke. For instance, knockdown of TRIM8 or TRIM47 has been shown to reduce infarct volume, suppress pro-inflammatory cytokines, and attenuate NF-κB activation in mouse stroke models [28,29]. Additionally, microglia-specific deletion of TRIM45 reduced production of pro-inflammatory cytokines and neuronal apoptosis by limiting K63-linked ubiquitination of TAB2, thereby inhibiting NF-κB activation [11]. Moreover, TRIM25 was up-regulated after ischemia and promoted ubiquitination of ATAD3A to drive PINK1/Parkin-dependent mitophagy, thereby aggravating ischemic brain injury [13]. TRIM9 alleviated ischemic injury by suppressing NF-κB-driven neuroinflammation. AAV-mediated TRIM9 up-regulation further reduced neuronal damage and improved recovery, particularly in aged mice [4]. By suppressing Keap1, TRIM16 augmented Nrf2/ARE signaling, thereby alleviating OGD/R-induced oxidative stress in hippocampal neurons [12]. In this study, we conducted a characterization of the phenotype associated with TRIM21 KO mice. We found that TRIM21 deficiency reduced infarct volumes, preserved BBB integrity, suppressed neuroinflammation, and improved neurological recovery. Furthermore, we confirmed an up-regulation of TRIM21 expression in astrocytes within cerebral ischemia models. In addition, MDA5 overexpression was found to rescue the phenotype of TRIM21 deficiency.

Neuroinflammation is a critical driver of pathological progression in ischemic stroke, markedly contributing to secondary brain injury and influencing long-term functional outcomes [30]. While microglia have traditionally been viewed as central mediators of neuroinflammation, emerging evidence indicates that astrocyte activation serves as a pivotal coordinator of post-ischemic inflammatory responses [31]. The functions of reactive astrocytes following ischemic stroke remain incompletely elucidated; nonetheless, they present a promising target for neurovascular protection post-stroke [32]. TRIM21 has been widely reported to play an essential role in a spectrum of disorders related to inflammation. For example, TRIM21 promotes post-myocardial infarction atrial remodeling and atrial fibrillation by activating the NF-κB–Nox2 pathway, leading to oxidative damage, inflammation, and connexin-43 dysregulation [14]. Moreover, TRIM21 neddylation promotes obesity-induced inflammation and metabolic disorders by destabilizing VHL and enhancing hypoxia-inducible factor-1α (HIF-1α)–IL-1β signaling in macrophages [33]. The role of TRIM21 in post-ischemic neuroinflammation, however, remained undefined. Here, we showed that TRIM21 expression was robustly elevated in astrocytes of the peri-infarct region at 72 h post-tMCAO. This astrocyte-specific up-regulation was recapitulated in an in vitro OGD/R model. Importantly, this temporal pattern of TRIM21 up-regulation corresponded closely with the established kinetics of reactive astrogliosis [34,35], suggesting its potential role in modulating astrocyte-mediated responses during the subacute phase of ischemic stroke [36]. Astrocyte activation, known as reactive astrogliosis, can induce secondary injury and is a critical driver of progressive neurological damage. Our findings demonstrated that TRIM21 deficiency exerted a neuroprotective effect in the tMCAO model. In contrast to WT mice, TRIM21^−/−^ mice exhibited smaller lesion volumes on MRI and improved short-term neurological outcomes. This effect was consistently observed in both female and male mice. Moreover, TRIM21 KO attenuated disruption of tight junction proteins, reduced EB extravasation, and decreased the number of TUNEL-positive cells, implying that TRIM21 contributes to BBB disruption and neuronal apoptosis. Astrocytes transition to a reactive state within the first week following ischemic insult, contributing to secondary injury through various mechanisms [37]. To elucidate underlying mechanisms, we performed transcriptomic analysis of ischemic hemispheres of WT and TRIM21^−/−^ mice at 72 h post-tMCAO. Our RNA-sequencing analysis revealed that TRIM21 deletion resulted in the down-regulation of genes associated with inflammatory responses. Furthermore, pathway enrichment analysis underscored the involvement of these genes in immune and oxidative stress pathways. We further validated these findings using ELISA, RT-qPCR, and oxidative stress assays, which demonstrated that TRIM21 deficiency suppressed the level of oxidative stress and pro-inflammatory cytokines in astrocytes after I/R. Taken together, TRIM21 plays a crucial role in amplifying astrocyte-mediated inflammation, thereby contributing to BBB disruption, neuronal apoptosis, and secondary injury following ischemic stroke. Our work also identified TRIM21 as a novel upstream regulator of this detrimental astrocytic response. The glial scar, primarily composed of reactive astrocytes, is often seen as a major barrier to neurite regeneration and the recovery of neurological function following ischemic stroke [38]. Future research will explore the role of TRIM21 in the formation of the glial scar following cerebral I/R injury.

MDA5, encoded by the IFIH1 gene, is a well-characterized cytosolic pattern recognition receptor integral to antiviral innate immunity. It recognizes viral double-stranded RNA and initiates downstream signaling through the adapter protein MAVS, leading to the activation of transcription factors IRF3 and NF-κB and subsequent production of type I interferons and pro-inflammatory cytokines [39]. However, its role in neuroinflammation following ischemic stroke has not yet been extensively investigated. This study provided the first evidence showing that MDA5 was up-regulated in astrocytes after cerebral ischemia and promoted neuroinflammation via direct interaction with TRIM21. Utilizing an integrated experimental approach including LC-MS/MS analysis, Co-IP, immunofluorescence, and SPR assays, we demonstrated a direct and enhanced physical interaction between TRIM21 and MDA5 under ischemic conditions. Mutational analysis revealed that the P-SPRY domain of TRIM21 was associated with the CARD domain of MDA5. Notably, both in vivo (tMCAO mouse models) and in vitro (OGD/R-treated astrocytes) analyses revealed concurrent up-regulation of MDA5 and TRIM21, predominantly localized in astrocytes within the peri-infarct region. This spatial and temporal correlation suggests that MDA5 may function beyond viral sensing, acting as a responsive mediator in ischemia-triggered neuroinflammation. Signaling through MDA5 molecules is modulated by posttranslational modifications [40]. Mechanistically, we identified that TRIM21 functions as an ubiquitin ligase, promoting K63-linked ubiquitination of MDA5. This posttranslational modification stabilized the MDA5 protein and facilitated the assembly of downstream signaling complexes, culminating in the phosphorylation of TBK1 and p65, and ultimately activating the NF-κB pathway. Consequently, this signaling cascade led to the transcriptional up-regulation and release of key proinflammatory cytokines, including IL-1β, IL-6, and TNF-α. The attenuated inflammatory response in TRIM21-deficient astrocytes further supported the critical role of this signaling axis. Our study is the first to reveal a direct functional link between TRIM21 and MDA5 in astrocytes and to elucidate the molecular pathway regulated via ubiquitination. However, the precise ubiquitination sites of MDA5 need to be further investigated. Current evidence indicates that MDA5 primarily recognizes long double-stranded RNA during viral infection; however, the exact mechanisms underlying its activation during cerebral ischemia remain unclear. Growing studies suggest that MDA5 can also be triggered by endogenous RNA species [41,42]. It is plausible that ischemic/hypoxic stress triggers the release of damage-associated molecular patterns (DAMPs), such as mitochondrial RNAs, which could serve as endogenous ligands facilitating MDA5 activation. Future studies will be needed to further understand this finding.

Small interfering RNA (siRNA) serves as a potent gene silencing tool widely employed to regulate target gene expression both in vivo and in vitro [43]. However, its therapeutic application remains challenging due to rapid enzymatic degradation and poor stability. These challenges are especially critical in the context of neurological disorders, where the BBB severely limits the penetration of most therapeutic agents. To overcome this barrier, receptor-mediated transcytosis has emerged as a promising targeting strategy. For example, Kumar et al. [44] identified a short peptide derived from the rabies virus glycoprotein (RVG) that specifically binds to the nicotinic acetylcholine receptor expressed on neuronal cells. Subsequent work by Liu et al. [45] leveraged this peptide as a targeting ligand to functionalize polyamidoamine (PAMAM) nanocarriers for enhanced brain delivery. This finding shifted the view of RVG29 to a BBB penetration enhancer, with its key mechanism being receptor-mediated transcytosis on brain capillary endothelial cells. Consequently, follow-up research has further developed RVG29 into a versatile brain-targeting peptide, primarily valued for its ability to enhance BBB penetration efficiency across diverse platforms. For instance, conjugating RVG29 to chimeric antigen receptor T (CAR-T) cells enhanced their BBB penetration and suppressed glioma progression in mouse models [46]. Similarly, an RVG29-based NP system delivered a METTL3 inhibitor for traumatic brain injury (TBI) intervention, improving brain delivery and demonstrating efficient internalization by microglia [26]. In light of these advancements, PLGA NPs represent an up-and-coming platform for siRNA delivery. U.S. Food and Drug Administration (FDA)-approved and renowned for their excellent biosafety profile, PLGA NPs can effectively encapsulate and protect nucleic acids from nuclease degradation, improve bioavailability, and allow for versatile surface modification for targeted delivery. Their well-established and scalable manufacturing process further facilitates the optimization of carrier properties for specific therapeutic applications [47]. Thus, RVG29-functionalized, siRNA-loaded PLGA NPs represent a promising strategy to overcome the key challenges in siRNA delivery. Herein, we constructed a revised RVG29 NP system aimed at delivering TRIM21-silencing therapy to the brain for ischemic stroke treatment. Our results demonstrated that siTRIM21@RVG-PLGA NPs significantly reduced ischemic lesion volume and enhanced functional recovery following stroke. Future work will aim to develop strategies that enhance cell-specific targeting.

In conclusion, we demonstrated in this study that TRIM21 positively regulated MDA5 protein stability through K63-linked polyubiquitination, which played a critical role in neuroinflammation and consequent cerebral I/R pathogenesis. Notably, we demonstrated that inhibiting the TRIM21–MDA5 axis could alleviate astrocyte-mediated inflammation, suggesting its potential as a therapy for ischemic brain injury.

## Materials and Methods

### Ethics statement

All animal procedures were conducted in accordance with the National Institutes of Health (NIH) Guide for the Care and Use of Laboratory Animals and were approved by the ethics committee of the Second Affiliated Hospital, Zhejiang University, China (approval no. AIRB-2022-082).

### Mice

All mice were maintained following NIH guidelines for animal care and were healthy, showing normal appearance and activity. Mice were housed under specific pathogen-free (SPF) conditions at 22 ± 2 °C with a 12-h light/dark cycle and provision of standard diet and water ad libitum. WT mice and Trim21 KO mice (NM-KO-190403) were obtained from Shanghai Model Organisms Center.

### Mouse tMCAO model

The tMCAO model was established based on previously described methods [48]. Mice (8 to 10 weeks old, 21 to 25 g) were anesthetized via isoflurane inhalation (3% for induction, 1.5% for maintenance). Body temperature was regulated at 37 ± 0.5 °C throughout surgery and recovery with a feedback-controlled heating pad. Middle cerebral artery occlusion (MCAO) was induced by introducing a silicone-coated nylon filament (RWD Life Science, MSMC21B100PK50) through the left external carotid artery (ECA) and advancing it into the internal carotid artery (ICA) to block the MCA. To confirm successful occlusion, cerebral blood flow (CBF) was measured using laser Doppler flowmetry (Moor Instruments, UK), requiring a drop to below 25% of the baseline value. After 60 min of obstruction, the filament was withdrawn to initiate reperfusion. Successful reperfusion was confirmed by cerebral blood flow returning to over 50% of the baseline. Sham-operated mice underwent identical procedures, excluding filament insertion. All surgical procedures were performed under aseptic conditions with efforts made to minimize suffering. Mice that failed to meet the CBF criteria mentioned above were excluded. Additionally, mice that could not eat or drink normally or fell into a coma after surgery were euthanized and excluded from the study. All surviving mice that exhibited neurological deficits upon recovery from anesthesia, that showed no evidence of hemorrhage (subarachnoid, skull base, or parenchymal) upon autopsy, and that had a confirmed cerebral infarct during sampling were included in the final experimental analysis. This study assessed the effects of TRIM21 KO, astrocytic MDA5 overexpression, and NP-mediated silencing on short-term neurological outcomes. The experimental design, group allocation, and animal numbers are summarized in Table S1.

### In vivo mouse brain MRI scanning

To evaluate ischemic brain injury, MRI was performed on day 3 after tMCAO using a 7.0-T small-animal magnetic resonance scanner (PharmaScan 7.0 T, Bruker). First, mice were anesthetized with isoflurane and positioned prone with the head secured in a radiofrequency transmit coil. Then, T2-weighted images were acquired with a 2-dimensional fast spin-echo sequence using the following parameters: repetition time (TR) = 2,500 ms, echo time (TE) = 35 ms, slice thickness = 0.55 mm, field of view (FOV) = 20 × 20 mm^2^, matrix size = 256 × 256. To correct for cerebral edema, infarct volume was expressed as a percentage and calculated using the following formula: Infarct volume (%) = (the contralateral hemisphere volume − non-infarct volume in the ipsilateral hemisphere)/(contralateral hemisphere volume × 2) × 100% [49].

### Neurological deficits and behavioral tests

All behavioral assessments were carried out by investigators blinded to experimental groups [50].

mNSS test: Neurological function was evaluated with the mNSS test on postoperative days 1, 3, 5, and 7, as previously described [51]. The mNSS test comprises motor, balance, and reflex subtests. Total scores range from 0 (no neurological deficit) to 14 (most severe impairment).

Rotarod test: Motor coordination and balance were assessed using an accelerating rotarod apparatus [52]. Motor performance was evaluated by placing mice on a rotarod that accelerated from 4 to 40 rpm over 300 s. The time until the mouse dropped from the rod (fall latency) was recorded. After 3 trials spaced 15 min apart, the average fall latency per mouse was computed for subsequent analysis.

Wire-hanging test: Forelimb motor coordination and grip strength were assessed using a wire-hanging test [53]. This apparatus consisted of a stainless-steel bar (50 cm long, 2 mm in diameter) positioned 37 cm above the bench surface between 2 vertical supports. Mice were placed at the midpoint of a horizontal bar for 4 consecutive trials lasting 30 s each. Performance was scored based on a 0 to 5 scale as follows: 0 (fell off), 1 (hung with 2 forepaws), 2 (attempted to climb with 2 forepaws), 3 (held on with forepaws and one/both hind paws), 4 (gripped with 4 limbs and tail wrapped around the bar), 5 (escaped to one support).

Adhesive removal test: Sensorimotor function was assessed using the adhesive removal test [54]. Briefly, a small adhesive tape patch (3 mm in diameter) was applied to the contralateral (impaired) forepaw. The mouse was then returned to its home cage, and the following parameters were recorded: (a) the latency to first contact with the tape (contact time), and (b) the latency to remove the adhesive completely (removal time).

### EB extravasation

The integrity of the BBB was evaluated using the EB extravasation method [55]. Mice undergoing tMCAO or sham operation at 72 h after surgery were injected with 2% EB (4 ml/kg, Sigma-Aldrich). The mice were euthanized 2 h after the injection and transcardially perfused with phosphate-buffered saline (PBS) to remove intravascular dye. The ipsilateral cerebral hemispheres (excluding olfactory bulbs and cerebellum) were collected, weighed, and homogenized in 1 ml of 50% trichloroacetic acid. After centrifugation at 12,000*g* for 20 min, the supernatant was collected. EB concentration was determined by measuring absorbance at 620 nm using a spectrophotometer and extrapolating from a standard curve. Results are expressed as micrograms of EB per gram of brain tissue (μg/g).

### TUNEL staining

TUNEL staining was performed on brain sections using a commercial TUNEL Assay Kit (Beyotime, China) in accordance with the manufacturer’s protocol. The percentage of cell apoptosis was quantified as the ratio of TUNEL-positive nuclei to 4′,6-diamidino-2-phenylindole (DAPI)-positive nuclei. Images were captured from the peri-infarct regions and quantitatively analyzed using ImageJ software.

### Cell culture

Primary neuron cells were isolated from embryonic day 17 (E17) mice as previously described [56]. The dissected cortices were dissociated enzymatically using 0.125% trypsin/EDTA and 0.025% deoxyribonuclease (DNase) I at 37 °C for 15 min. After digestion, the cells were centrifuged, resuspended, filtered through a 70-μm cell strainer, and plated in Neurobasal medium supplemented with B27. Cultured neurons were maintained at 37 °C in a 5% CO_2_ atmosphere and used for experiments at 7 to 10 d in vitro (DIV).

Primary astrocytes and microglia cultures were prepared from cerebral cortices of postnatal day 1 to 2 C57BL/6J mice as previously described [57]. The cerebral cortices were dissected, mechanically dissociated, and digested with 0.125% trypsin/EDTA and 0.025% DNase I at 37 °C for 15 min. The cell pellet was resuspended after being centrifuged at 1,000 rpm for 5 min, filtered using a 70-μm strainer, and then seeded into T75 flasks. Cells were maintained in high-glucose Dulbecco’s modified Eagle’s medium (DMEM) supplemented with 10% fetal bovine serum (FBS) at 37 °C under 5% CO₂. After reaching confluence, microglia were isolated by shaking the flasks at 200 rpm for 1 h. The remaining adherent astrocytes were harvested by trypsinization after 10 to 14 DIV.

### Plasmids and siRNA transfection

The HA-TRIM21-related plasmids, Flag-MDA5-related plasmids, and Myc-ubiquitin-related plasmids were purchased from Youbio Biological Technology Company (Hunan, China). The siRNA was purchased from GenePharma Biological Technology (Shanghai, China), and siRNA sequences targeting mouse MDA5 are sense 5′-GGGCGAUGUAAAGAAAUCUTT-3′ and antisense 5′-AGAUUUCUUUACAUCGCCCTT-3′. Plasmid and siRNA transfection were performed using Lipofectamine 2000 (Invitrogen, Carlsbad, CA, USA) according to the manufacturer’s protocol.

### Oxygen–glucose deprivation and reoxygenation

The OGD/R model was established as previously described [57]. Cultured cells were washed and immersed in glucose- and FBS-free DMEM, then placed in a hypoxic chamber (Thermo Scientific, USA) filled with a pre-mixed gas (95% N_2_, 5% CO_2_) at 37 °C. After OGD exposure, cells were returned to a normoxic incubator (95% air, 5% CO_2_) and replenished with normal culture medium. Control cells were maintained in normal medium under normoxic conditions for an equivalent duration. Due to the differential sensitivity of astrocytes, microglia, and neurons to OGD, the insult duration was optimized as 6, 3, and 2 h, respectively. Reoxygenation time was adjusted according to cell type and experimental requirements.

### Immunofluorescence staining

Cultured cells were fixed in 4% paraformaldehyde (PFA) for 20 min at room temperature and rinsed in PBS. Following fixation in 4% PFA (24 h, 4 °C), brains were dehydrated in a graded sucrose series (15% and 30%) at 4 °C until sinking, embedded in optimal cutting temperature compound (OCT), and coronally sectioned at 16 μm on a Leica cryostat microtome. For cultured cells and brain sections, samples were equilibrated to room temperature. After appropriate permeabilization, nonspecific sites were blocked using 10% normal donkey serum in PBS for 1 h. The cells and tissue sections were then probed with primary antibodies overnight at 4 °C, followed by incubation with corresponding species-matched secondary antibodies for 1 h at room temperature, protected from light. DAPI (Thermo Scientific, USA) stained the nucleus, and anti-fluorescence quenching tablets were used to seal the slides. All images were captured on a Leica DMi8 confocal microscope with identical acquisition parameters maintained within each experiment.

### Western blot

The procedure for Western blot analysis was followed as previously detailed [58]. In brief, the extraction of proteins from tissues and cells was performed using radioimmunoprecipitation assay (RIPA) containing protease and phosphatase inhibitors, followed by 30-min incubation on ice and centrifugation to collect the supernatant. Protein concentrations were measured with a BCA assay kit (Thermo Scientific, USA). After denaturation, equal protein amounts were separated by sodium dodecyl sulfate–polyacrylamide gel electrophoresis (SDS-PAGE) and electrotransferred onto polyvinylidene difluoride (PVDF) membranes (Millipore, Germany). After transfer, the membranes were blocked with 5% nonfat milk prepared in Tris-buffered saline with Tween-20 (TBST) for 1 h at room temperature. Subsequently, they were probed with appropriate primary antibodies overnight at 4 °C, followed by 3 washes with TBST and a 1-h incubation with secondary antibodies at room temperature. Signals were detected by enhanced chemiluminescence (Millipore, Germany) and quantified using ImageJ software (NIH, USA).

### Data collection and preprocessing

RNA-sequencing transcriptome data from the cortex (GSE112348) and hippocampus (GSE202391) of mice subjected to tMCAO or sham surgery were retrieved from the GEO database (http://www.ncbi.nlm.nih.gov/geo). Differential gene expression analysis between tMCAO and sham groups was performed in R Studio using the following thresholds: |Log₂(fold change)| > 0.58 and adjusted *P* value < 0.05. The single-cell RNA sequencing data are publicly available from the GEO, accession number GSE227651.

### RNA sequencing

At 72 h post-tMCAO, total RNA was isolated from the ischemic hemispheres of WT and TRIM21^−/−^ mice using TRIzol (Thermo Scientific, USA) and processed for RNA sequencing. The quality of RNA was evaluated using the Agilent 2100 Bioanalyzer and verified by RNase-free agarose gel electrophoresis. Eukaryotic mRNA was enriched by oligo (dT) beads, fragmented, and reverse-transcribed into cDNA using the NEBNext Ultra RNA Library Prep Kit for Illumina (NEB #7530). The resulting double-stranded cDNA was end-repaired, adenylated, and ligated to Illumina adapters. Purification was performed with AMPure XP Beads (1.0X), followed by PCR amplification. Libraries were sequenced on an Illumina NovaSeq 6000 platform (Gene Denovo Biotechnology Co., Guangzhou, China). The sequencing and bioinformatics analysis were provided by Gene Denovo Biotechnology Company (Guangzhou, China).

### Enzyme-linked immunosorbent assay

Levels of the pro-inflammatory cytokines (IL-1β, IL-6, and TNF-α) in tissue homogenates and astrocyte culture supernatants were measured with mouse ELISA kits according to the manufacturer’s manuals (Youke Life Science Technology Co., Hangzhou, China).

### Determination of the levels of oxidative stress

Intracellular ROS in astrocytes were detected by incubating cells with 10 μM DCFH-DA (2',7'-dichlorodihydrofluorescein diacetate, Solarbio, China) in complete medium for 30 min at 37 °C in the dark. After PBS washing to remove excess probe, fluorescence was imaged using a fluorescence microscope. Levels of key oxidative stress parameters—MDA, SOD, and GSH-Px—were quantified in both brain tissues and cultured astrocytes using corresponding kits from Nanjing Jiancheng Bioengineering Institute, following the manufacturer’s protocols.

### Reverse transcription quantitative real-time PCR

Following isolation with TRIzol, total RNA was reverse-transcribed into cDNA using the PrimeScript RT kit (Takara, Japan). The resulting cDNA was amplified for qPCR analysis using SYBR Green PCR Master Mix (Takara) on a StepOnePlus Real-Time PCR System (Applied Biosystems, USA) in accordance with the standard protocol. Furthermore, the 2^−ΔΔCt^ method was applied to calculate the expression of each gene, normalized to β-actin. The sequences of the primers used for RT-qPCR are listed in Table 1.

**Table 1.** Sequences of the primers

Genes of interest	Primers
β-actin	5′-GTGACGTTGACATCCGTAAAGA-3′ 5′-GCCGGACTCATCGTACTCC-3′
IL-1β	5′-GCAACTGTTCCTGAACTCAACT-3′ 5′-ATCTTTTGGGGTCCGTCAACT-3′
IL-6	5′-TACCACTTCACAAGTCGGAGGC-3′ 5′-CTGCAAGTGCATCATCGTTGTTC-3′
TNF-α	5′-CAGGCGGTGCCTATGTCTC-3′ 5′-CGATCACCCCGAAGTTCAGTAG-3′

### Co-IP and mass spectrometry analysis

Cells or tissues were lysed in ice-cold IP lysis buffer (Thermo Scientific, USA) supplemented with protease and phosphatase inhibitors. After centrifugation, the supernatant was collected and incubated with the appropriate primary antibody overnight at 4 °C with gentle rotation. Protein A/G magnetic beads (Thermo Scientific, USA) were used to capture the immune complexes for 2 h at room temperature, followed by 3 washes with ice-cold wash buffer. For Western blot analysis, complexes were eluted in 2× SDS loading buffer by boiling at 95 °C for 5 min, followed by brief centrifugation to collect the supernatant. The obtained supernatant was utilized for subsequent Western blot analysis. For proteomic analysis, the washed bead-bound complexes were subjected to mass spectrometry by Shanghai Bioprofile Technology. The resulting LC-MS/MS data were processed using MaxQuant (v.2.4.14.0).

### Molecular docking

The UniProt database (https://www.uniprot.org/) was used to obtain the amino acid sequences for MDA5 (UniProt: Q9BYX4) and TRIM21 (UniProt: P19474). The 3-dimensional complex structure of MDA5 and TRIM21 was predicted using AlphaFold3 (https://www.alphafoldserver.com) under a 1:1 molar ratio. The highest-confidence model returned by the server was selected and visualized using PyMOL (v.2.3.0) for figure generation and interface inspection.

### SPR assay

To investigate the direct interaction between MDA5 and TRIM21 and to map the interacting domains, the SPR experiment was carried out utilizing a Biacore 8K system (Cytiva). Recombinant full-length MDA5 or its truncated variant (lacking the CARD domain) was immobilized on a CM5 sensor chip. A dilution series of full-length TRIM21 or its truncated variant (lacking the P-SPRY domain) was injected at a flow rate of 20 μl/min. The association phase was monitored for 100 s, followed by a 180-s dissociation phase in the analyte buffer. A total of 7 analyte concentrations were tested in ascending order. The sensor chip surface was regenerated after each concentration cycle. Sensorgram data were processed using Biacore Insight Evaluation Software to obtain binding curves and calculate affinity constants.

### SDD-AGE

MDA5 oligomerization and MAVS aggregation were analyzed by SDD-AGE as previously described [59]. Briefly, cells were harvested and lysed by grinding in a buffer containing 10 mM tris–HCl (pH 7.5), 1.5 mM MgCl_2_, 10 mM KCl, and 0.25 M d-mannitol supplemented with a protease inhibitor cocktail. The lysates were cleared by centrifugation at 700*g* and 4 °C for 10 min. The supernatant was then centrifuged at 10,000*g* and 4 °C for 30 min to separate the crude mitochondrial fraction and cytosolic extracts. The crude mitochondria extract was resuspended in 1× sample buffer [0.5× Tris–borate–EDTA (TBE), 10% (v/v) glycerol, 2% (w/v) SDS, and 0.0025% (w/v) bromophenol blue] and loaded onto a 1.5% agarose gel (1× TBE, 0.1% SDS) at 80 V for 90 min at 4 °C. Finally, samples were transferred onto a PVDF membrane and subjected to immunoblot analysis using the indicated antibodies.

### AAV delivery

To achieve astrocyte-specific overexpression of MDA5, TRIM21^−/−^ mice received stereotaxic intracranial injections of AAV2/5 vectors encoding GfaABC1D-Ifih1-3×FLAG (AAV-MDA5, 3.5 × 10^12^ vg/ml) or GfaABC1D-EGFP-P2A-3×FLAG (AAV-Ctrl, 1.8 × 10^13^ vg/ml) (Obio Technology, China). Using a 10-μl syringe fitted with a 26-gauge needle (Hamilton, 7642-01, 7803-07), 1 μl of viral suspension was injected into each of the following stereotaxic coordinates in the left hemisphere at a rate of 200 nl/min: the hippocampal CA1 region [anteroposterior (AP): −2.00 mm, mediolateral (ML): −1.55 mm, dorsoventral (DV): −1.55 mm], cerebral cortex (AP: 0.00 mm, ML: −2.05 mm, DV: −1.50 mm), and striatum (AP: 0.00 mm, ML: −2.05 mm, DV: −3.50 mm) [11]. Mice were utilized 3 weeks after AAV injection.

### Astrocyte isolation

Mice were euthanized and transcardially perfused with ice-cold PBS. The brain tissues were rapidly dissected, minced into fine pieces, and subjected to enzymatic digestion. Tissue fragments were digested at 37 °C for 30 min in RPMI 1640 medium supplemented with 2 mM l-glutamine, containing 0.125% trypsin–EDTA, 0.2% collagenase type III, and 3 U/ml dispase [60]. After adding DNase I to the tissues, they were incubated for 15 min, and the digestion was neutralized with PBS and 10% FBS. The digested tissues were filtered through a 70-μm strainer and centrifuged. The cell pellet was resuspended in a 30% Percoll solution and subjected to density gradient centrifugation to remove myelin debris. The single-cell suspension was collected for subsequent use. The samples were first incubated with an Fc receptor blocking reagent and then incubated with Anti-ACSA-2 MicroBeads (Miltenyi Biotec) at 4 °C for 15 min. The labeled cell suspension was passed through a separator column (Miltenyi Biotec) placed within a magnetic separation frame. Following washing, the magnetically retained ACSA-2+ astrocytes were eluted as the purified cell fraction for subsequent analysis.

### Preparation and characterization of PLGA-RVG29 NPs

PLGA NPs were synthesized using a solvent evaporation technique. Briefly, 100 μg of siTRIM21 (5′-GCCCAGAAUACCAAGAAGATT-3′ and 5′-UCUUCUUGGUAUUCUGGGCTT-3′) in TE buffer was emulsified via sonication (30 s, ice bath) in 1 ml of dichloromethane (DCM) containing PLGA20K (4 mg), PLGA20K-mPEG2K (4 mg), and PLGA20K-mPEG5K-Mal (2 mg). Further emulsification of the primary emulsion was achieved by sonication for 30 s in an ice bath, using 2 ml of a 1% (w/v) polyvinyl alcohol (PVA) solution. Then, the emulsion was diluted in a 0.5% PVA solution under continuous stirring for 1 min. The organic solvent was evaporated using a rotary evaporator for 3 h. The NPs were purified via 3 cycles of PBS washing and centrifugation (14,000 rpm, 20 min), then resuspended in 2 ml PBS. To conjugate RVG29 (rabies virus glycoprotein peptide 29) to the NPs, PLGA NPs were exposed to 2 mg of RVG29 peptide for 12 h at 4 °C. Unbound peptide was removed by centrifugation and PBS washes.

The size distribution and morphology of PLGA-RVG29 NPs were characterized by dynamic light scattering (Malvern Zetasizer Lab, UK) and transmission electron microscopy (JEOL JEM-1400 Flash, Japan), respectively.

### In vivo brain-targeting evaluation

To evaluate delivery to the brain, mice received an intravenous injection of 100 μl of PLGA-RVG29 NPs, which included 1 mg/kg siRNA and 2 mg/kg DiR. Biodistribution was monitored at 4, 8, 12, and 24 h post-injection using an in vivo imaging system (IVIS Spectrum, PerkinElmer, USA). At 24 h post-injection, the mice were euthanized, and major organs (the brain, heart, liver, spleen, lung, and kidney) were harvested for ex vivo fluorescence imaging and further analysis.

### In vivo safety evaluation

Healthy mice were intravenously injected with PBS or siTRIM21@RVG-PLGA NPs. At days 1 and 7 post-injection, whole blood and major organs, including the brain, heart, liver, spleen, lung, and kidney, were collected. The concentrations of AST, ALT, BUN, and CRE were measured, along with counts of RBCs, WBCs, PLT, and NEU, following the manufacturer’s instructions. Moreover, H&E staining was conducted on major organs for histological assessment of morphological alterations.

### Statistical analysis

Data are expressed as mean ± SD from at least 3 separate experiments. All statistical analyses were performed using GraphPad Prism (version 10.0). The normality of data distribution was assessed using the Shapiro–Wilk test. For comparisons between 2 groups, homogeneity of variances was assessed using the *F* test. For comparisons among 3 or more groups, homogeneity of variances was assessed using Bartlett’s test. An unpaired *t* test was employed for comparing 2 groups of normally distributed data with equal variance. One-way analysis of variance (ANOVA) was used for comparing 3 or more groups under identical conditions, followed by Tukey’s post hoc test for multiple comparisons. If the equal variance assumption was violated, Welch’s ANOVA with Dunnett’s T3 post hoc test was utilized for comparisons among multiple groups. Furthermore, a Welch’s *t* test was applied to compare 2 groups. For data that did not follow a normal distribution, comparisons between 2 groups were conducted using the Mann–Whitney *U* test, while the Kruskal–Wallis test was employed for comparisons involving 3 or more groups, followed by Dunn’s post hoc test with Bonferroni correction. A 2-way ANOVA with Tukey’s post hoc test was employed to examine the recovery of neurological functions. A *P* value of less than 0.05 was considered statistically significant.

## Data Availability

The datasets used and/or analyzed in the current study are available from the corresponding authors on reasonable request.

## Supplementary Materials

Supplementary 1 Graphical Abstract Figs. S1 to S7 Table S1
