# Supplementary material for: TRIM21 Exacerbates Ischemic Brain Injury by Promoting Astrocyte-Mediated Neuroinflammation via K63-Linked Ubiquitination of MDA5
Source: Research (Wash D C). 2026 Mar 17;9:1200. doi: 10.34133/research.1200 (PMC12992933; doi:10.34133/research.1200)
Supplement: Supplementary 1 — Graphical Abstract Figs. S1 to S7 Table S1 [file research.1200.f1.zip › Supplementary Table 1.docx]

**Supplementary Table 1. Experimental Design, Group Allocation, and Number of Mice Utilized.**

|  | Total | Excluded***** | Dead****** | Mortality Rate (%) | Survived | Usage |
| --- | --- | --- | --- | --- | --- | --- |
| **Experiment 1** | | | | | | |
| WT, (sham) | 14 | 0 | 0 | 0 | 14 | 1. MRI assessment of cerebral infarct volume (n = 8/group).  2. All of the mice were used for behavioral tests. |
| KO, (sham) | 14 | 0 | 0 | 0 | 14 |  |
| WT, (tMCAO) | 15 | 1 | 5 | 35.7 | 9 |  |
| KO, (tMCAO) | 15 | 1 | 3 | 21.4 | 11 |  |
| **Experiment 2** | | | | | | |
| WT, (sham，female) | 11 | 0 | 0 | 0 | 11 | 1. MRI assessment of cerebral infarct volume (n = 8/group).  2. All of the mice were used for mNSS test. |
| KO, (sham，female) | 11 | 0 | 0 | 0 | 11 |  |
| WT, (tMCAO，female) | 16 | 1 | 4 | 26.7 | 11 |  |
| KO, (tMCAO，female) | 14 | 1 | 2 | 15.4 | 11 |  |
| **Experiment 3** | | | | | | |
| KO + AAV-Control + tMCAO | 13 | 0 | 2 |  | 11 | 1. MRI assessment of cerebral infarct volume (n = 8/group), among whom 5 mice/group were used for proinflammatory cytokines detection.  2. All of the mice were used for mNSS test. |
| KO + AAV-MDA5 + tMCAO | 15 | 1 | 3 | 21.4 | 11 |  |
| **Experiment 4** | | | | | | |
| WT, (sham) | 10 | 0 | 0 | 0 | 10 | 1. MRI assessment of cerebral infarct volume in tMCAO groups (n = 8/group).  2. All of the mice were used for behavioral tests. |
| tMCAO + siNC@RVG-PLGA NPs | 14 | 2 | 4 | 33.3 | 8 |  |
| tMCAO + siTRIM21@RVG-PLGA NPs | 12 | 0 | 3 | 25 | 9 |  |

* Failure to meet cerebral blood flow (CBF) criteria

** Due to severe cerebral edema and brain herniation
