# Supplementary material for: TRIM21 Exacerbates Ischemic Brain Injury by Promoting Astrocyte-Mediated Neuroinflammation via K63-Linked Ubiquitination of MDA5
Source: Research (Wash D C). 2026 Mar 17;9:1200. doi: 10.34133/research.1200 (PMC12992933; doi:10.34133/research.1200)
Supplement: Supplementary 1 — Graphical Abstract Figs. S1 to S7 Table S1 [file research.1200.f1.zip › Supplementary Material Figure Caption.docx]

**Supplementary Fig. 1. TRIM21 knockout (TRIM21^−/−^) mice are generated and validated.** (**A**) Knockout strategy chart for TRIM21. (**B**) Representative image of genotyping of WT and TRIM21^−/−^ mice by PCR using genomic DNA extracted from newborn mouse tails. (**C**) Representative western blot of TRIM21 protein expression in brain tissues of WT and TRIM21^−/−^ mice.

**Supplementary Fig. 2.** (**A-B**) TRIM21 deficiency reduced cerebral infarct volume (n = 8, unpaired t-test) and improved neurological function (n = 11, unpaired t-test) in the female mice. All data are expressed as means ± SD. ****p* < 0.001 vs. indicated groups.

**Supplementary Fig. 3.** (**A**) Quantitative analysis of mean fluorescence intensity of TRIM21 in astrocytes, microglia, and neurons within the peri-infarct region of the ischemic mice. (n = 5, unpaired t-test). (**B**) UMAP plot visualizing and unsupervised clustering of all cells colored by cell types. (**C**) Dot plot representing the expression level of cell-type-specific genes within each cluster. (**D**) Violin plot showing Trim21 expression levels across cell subclusters where its expression was detected, by Mann-Whitney U test. All data are expressed as means ± SD. ***p* < 0.01 and ****p* < 0.001 vs. indicated groups.

**Supplementary Fig. 4.** (**A**) RT-qPCR analysis of IL-1β, IL-6, and TNF-α mRNA levels in ischemic hemispheres of WT and TRIM21^−/−^ mice on day 3 post-tMCAO. (n = 5, Welch’s ANOVA followed by Dunnett’s T3 post hoc test). (**B**) RT-qPCR analysis of proinflammatory cytokine mRNA levels in OGD/R-treated WT and TRIM21^−/−^ primary astrocytes. (n = 5, Welch’s ANOVA followed by Dunnett’s T3 post hoc test). All data are expressed as means ± SD. **p* < 0.05, ***p* < 0.01, and ****p* < 0.001 vs. indicated groups.

**Supplementary Fig. 5.** (**A**) Western blot images and quantification of TBK1 and P65 phosphorylation in MDA5-knockdown WT primary astrocytes after OGD/R. (n = 5, Welch’s ANOVA followed by Dunnett’s T3 post hoc test). (**B**) Endogenous MDA5 oligomerization and MAVS aggregation in WT and TRIM21^−/−^ primary astrocytes after OGD/R, assessed by SDD-AGE and IB with anti-MDA5 and anti-MAVS. Whole cell lysates were further analyzed by SDS-PAGE and probed by IB with the indicated antibodies. Results are representative of three independent experiments. All data are expressed as means ± SD. **p* < 0.05, ***p* < 0.01, and ****p* < 0.001 vs. indicated groups.

**Supplementary Fig. 6.** (**A**) Western blot analysis of MDA5 expression in astrocytes isolated from the brain tissues of mice injected with AAV-MDA5 or AAV-Control. Astrocytes were isolated using ACSA-2+ microbeads. (n = 5, unpaired t-test). (**B**) *Ex vivo* images of main organs at 24 h following intravenous injection of DiR-labeled NPs. (**C**) Immunofluorescence images showing co-localization of DiO-conjugated NPs (green) with the neuronal marker NeuN (red, left) and the microglia marker Iba1 (red, right) at 24 h post-injection. Quantitative analysis was performed together with Fig. 8F. (n = 5, one-way ANOVA followed by Tukey’s post hoc test). (**D**) Western blot analysis of TRIM21 expression in astrocytes isolated from the brain tissues of mice injected with siTRIM21@RVG-PLGA NPs or siNC@RVG-PLGA NPs. Astrocytes were isolated using ACSA-2+ microbeads. (n = 5, unpaired t-test). All data are expressed as means ± SD. ****p* < 0.001 vs. indicated groups.

**Supplementary Fig. 7.** **Safety evaluation in vivo.** (**A**) Routine blood indexes of red blood cells (RBC), white blood cells (WBC), platelets (PLT), and neutrophils (NEU) on day 1 and day 7 after injections of siTRIM21@RVG-PLGA NPs compared with healthy mice. (n = 8, one-way ANOVA followed by Tukey’s post hoc test). (**B**) Blood biochemical parameters of aspartate aminotransferase (AST), alanine aminotransferase (ALT), blood urea nitrogen (BUN), and creatinine (CRE) on day 1 and day 7 after injections of siTRIM21@RVG-PLGA NPs compared with healthy mice. (n = 8, one-way ANOVA followed by Tukey’s post hoc test). (**C**) Representative H&E staining of the brain, heart, liver, kidney, spleen, and lung on day 1 and day 7 after injections of siTRIM21@RVG-PLGA NPs compared with healthy mice. Scale bars: 100 μm. All data are expressed as means ± SD.
