# Supplementary figures and images for: TRIM21 Exacerbates Ischemic Brain Injury by Promoting Astrocyte-Mediated Neuroinflammation via K63-Linked Ubiquitination of MDA5
Source: Research (Wash D C). 2026 Mar 17;9:1200. doi: 10.34133/research.1200 (PMC12992933; doi:10.34133/research.1200)

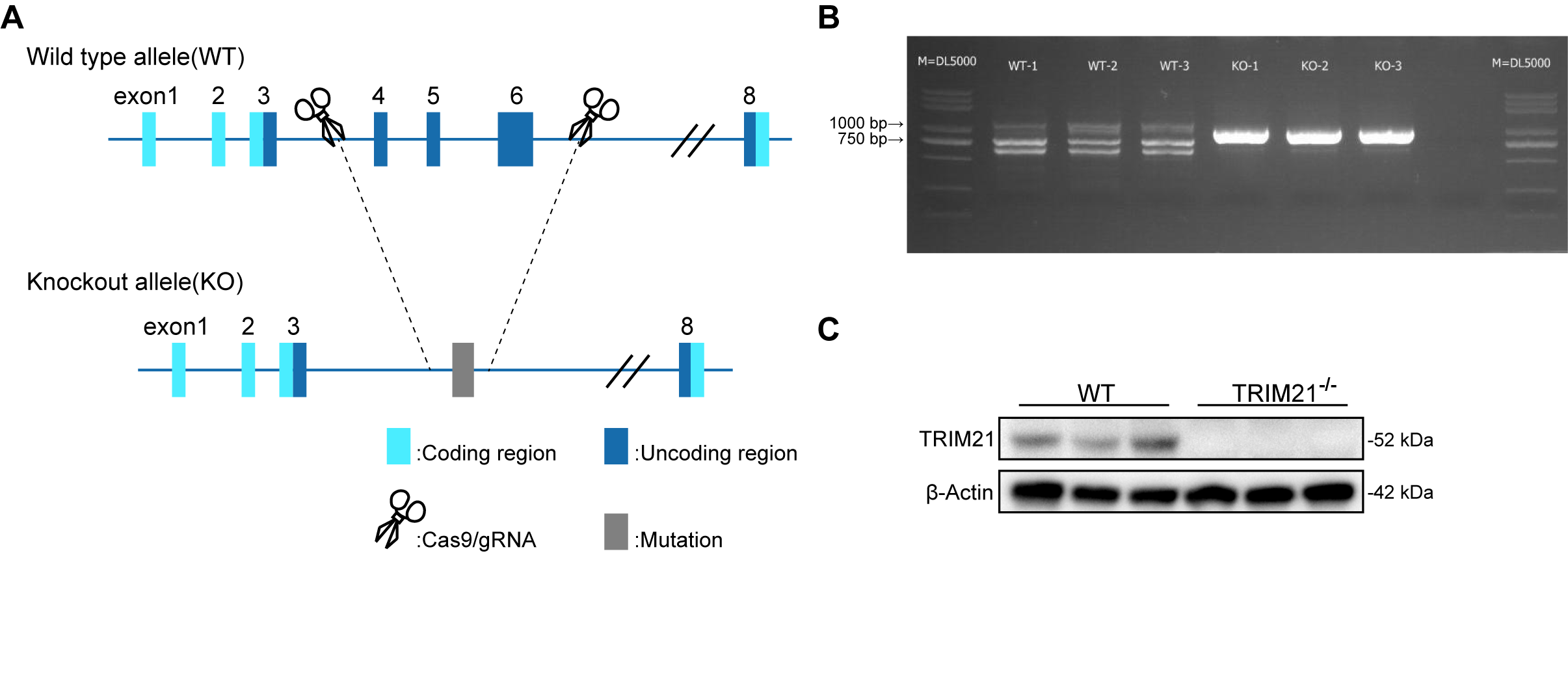

Supplement: Supplementary 1 — Graphical Abstract Figs. S1 to S7 Table S1 [file research.1200.f1.zip › updated-Supplementary Fig. 1.tif]

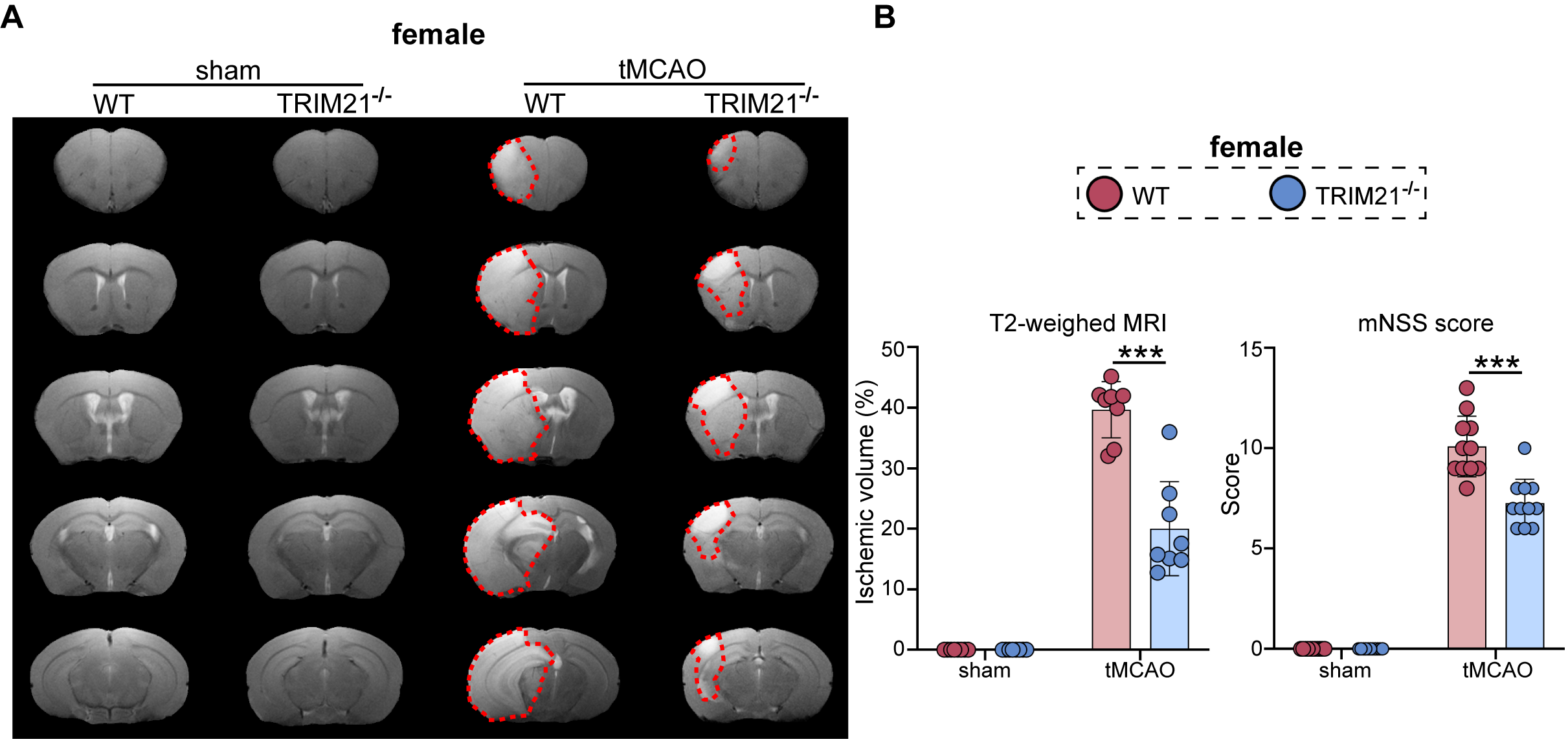

Supplement: Supplementary 1 — Graphical Abstract Figs. S1 to S7 Table S1 [file research.1200.f1.zip › updated-Supplementary Fig. 2.tif]

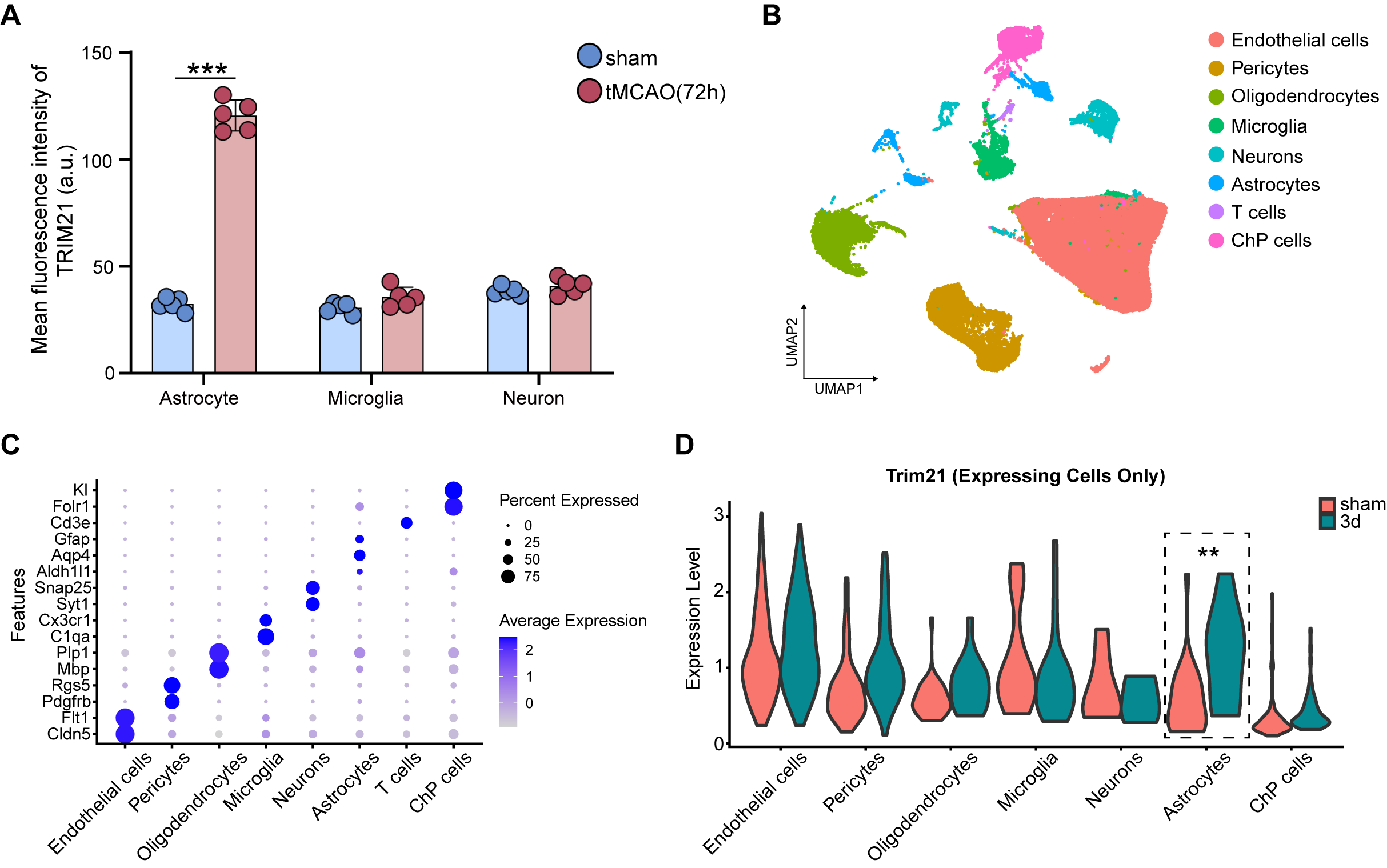

Supplement: Supplementary 1 — Graphical Abstract Figs. S1 to S7 Table S1 [file research.1200.f1.zip › updated-Supplementary Fig. 3.tif]

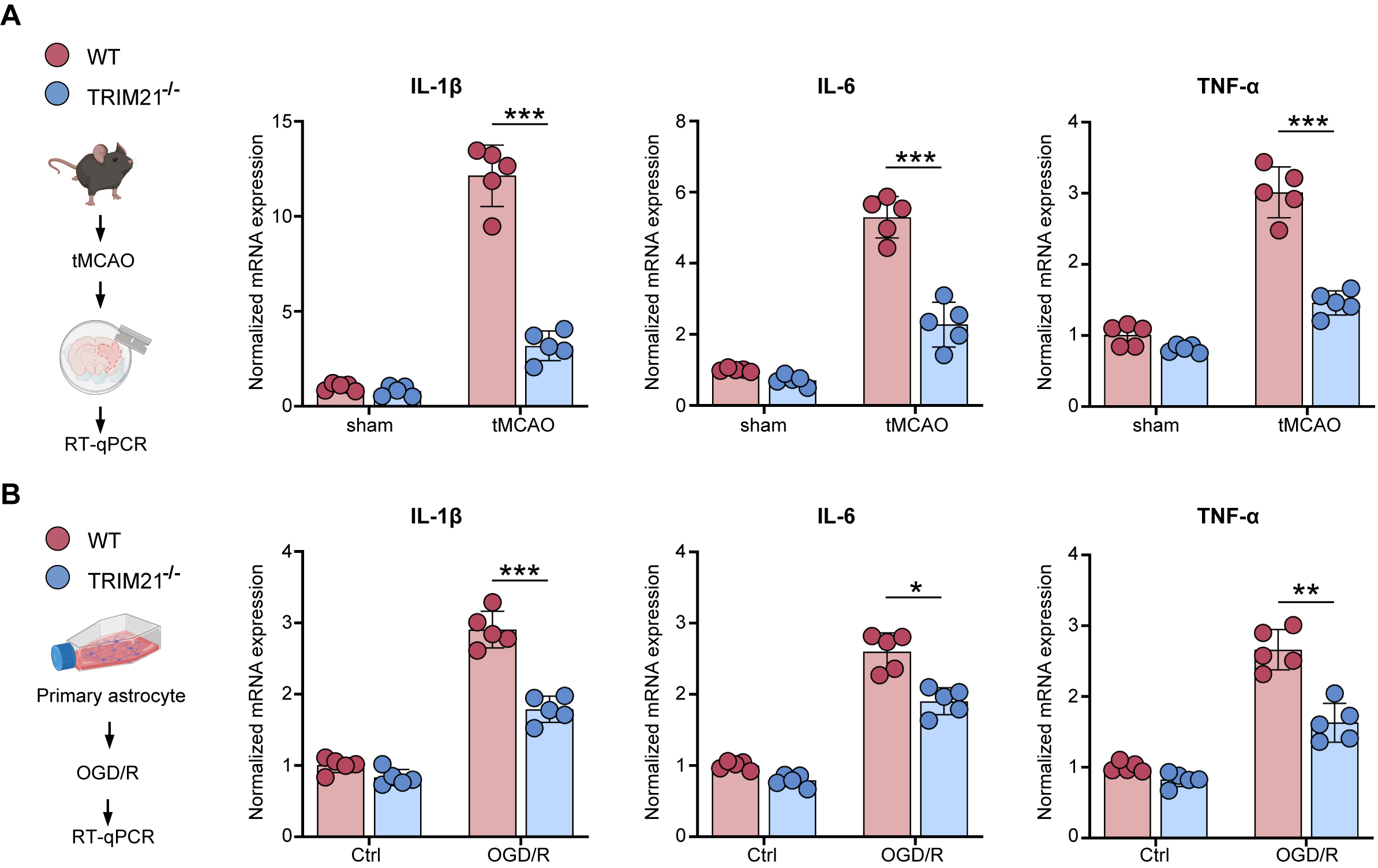

Supplement: Supplementary 1 — Graphical Abstract Figs. S1 to S7 Table S1 [file research.1200.f1.zip › updated-Supplementary Fig. 4.tif]

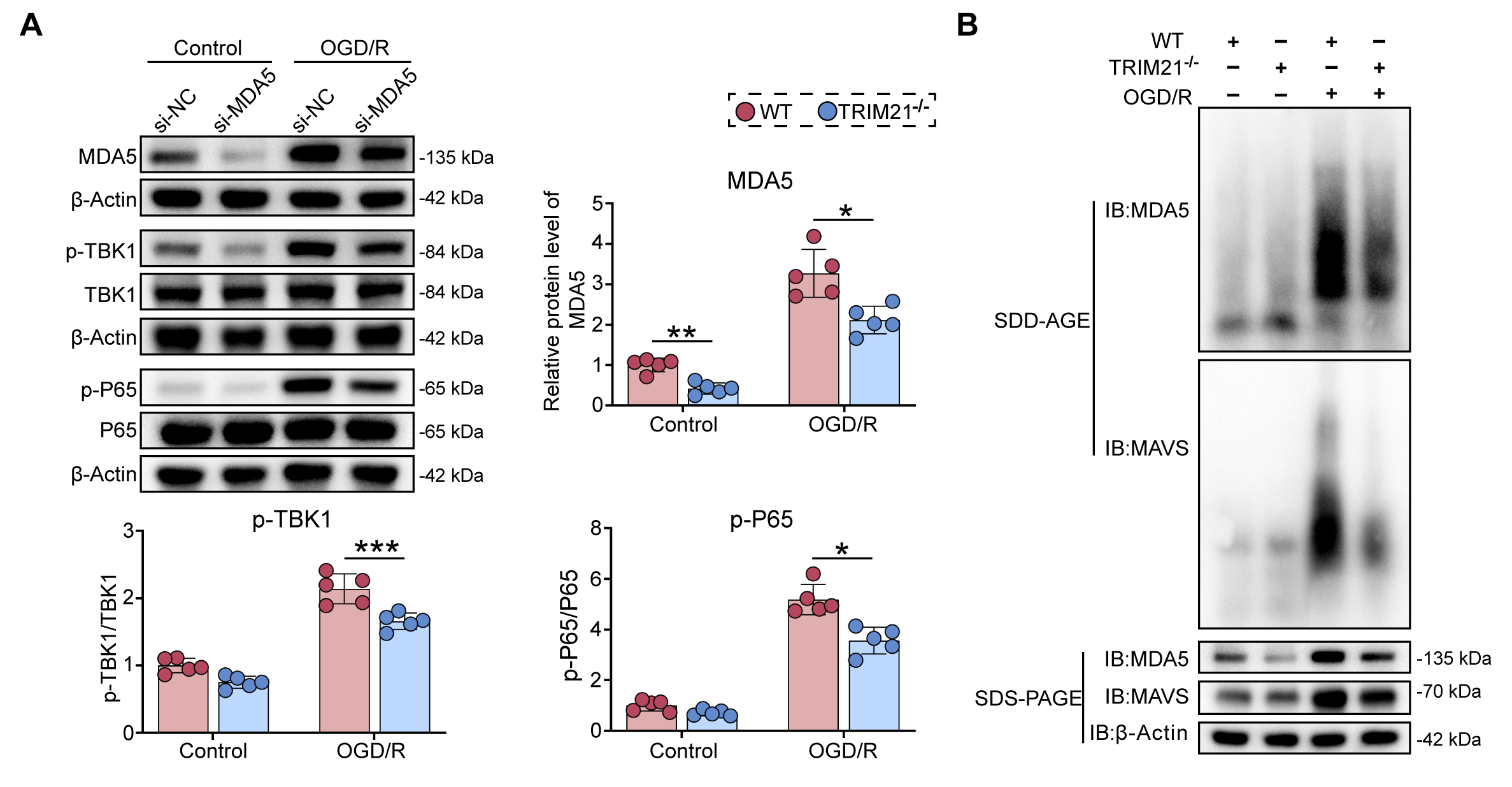

Supplement: Supplementary 1 — Graphical Abstract Figs. S1 to S7 Table S1 [file research.1200.f1.zip › updated-Supplementary Fig. 5.tif]

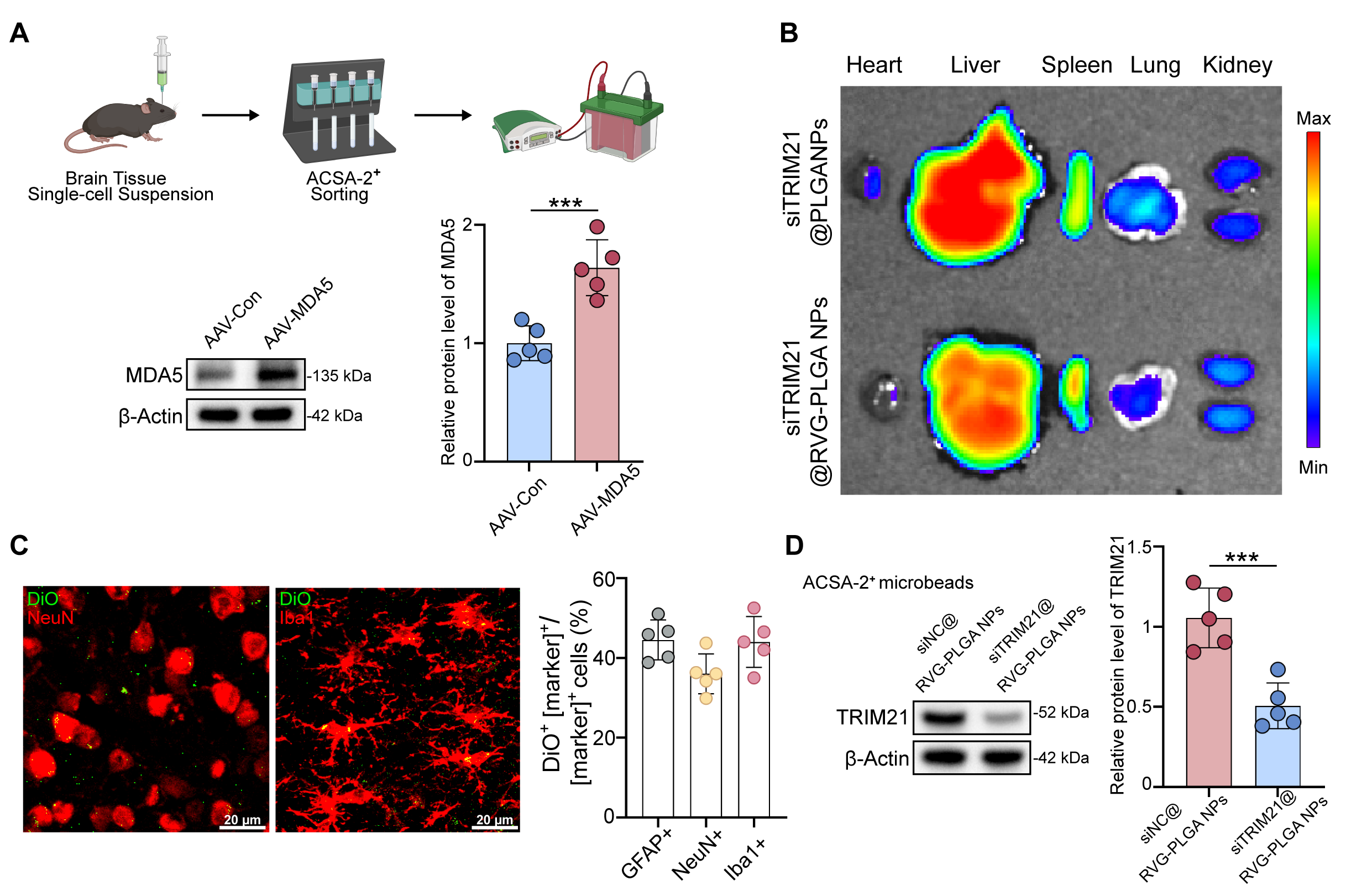

Supplement: Supplementary 1 — Graphical Abstract Figs. S1 to S7 Table S1 [file research.1200.f1.zip › updated-Supplementary Fig. 6.tif]

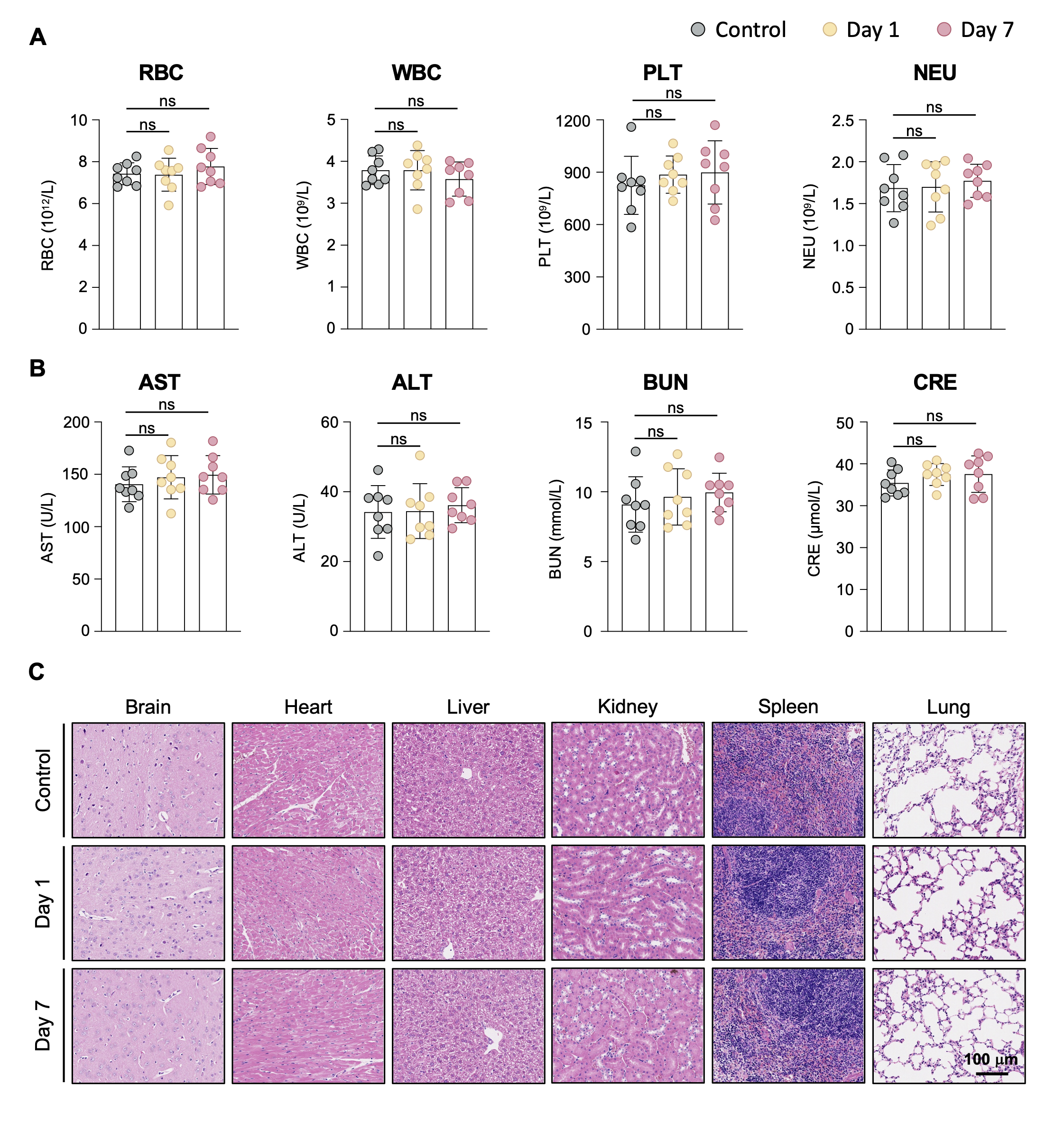

Supplement: Supplementary 1 — Graphical Abstract Figs. S1 to S7 Table S1 [file research.1200.f1.zip › updated-Supplementary Fig. 7.tiff]

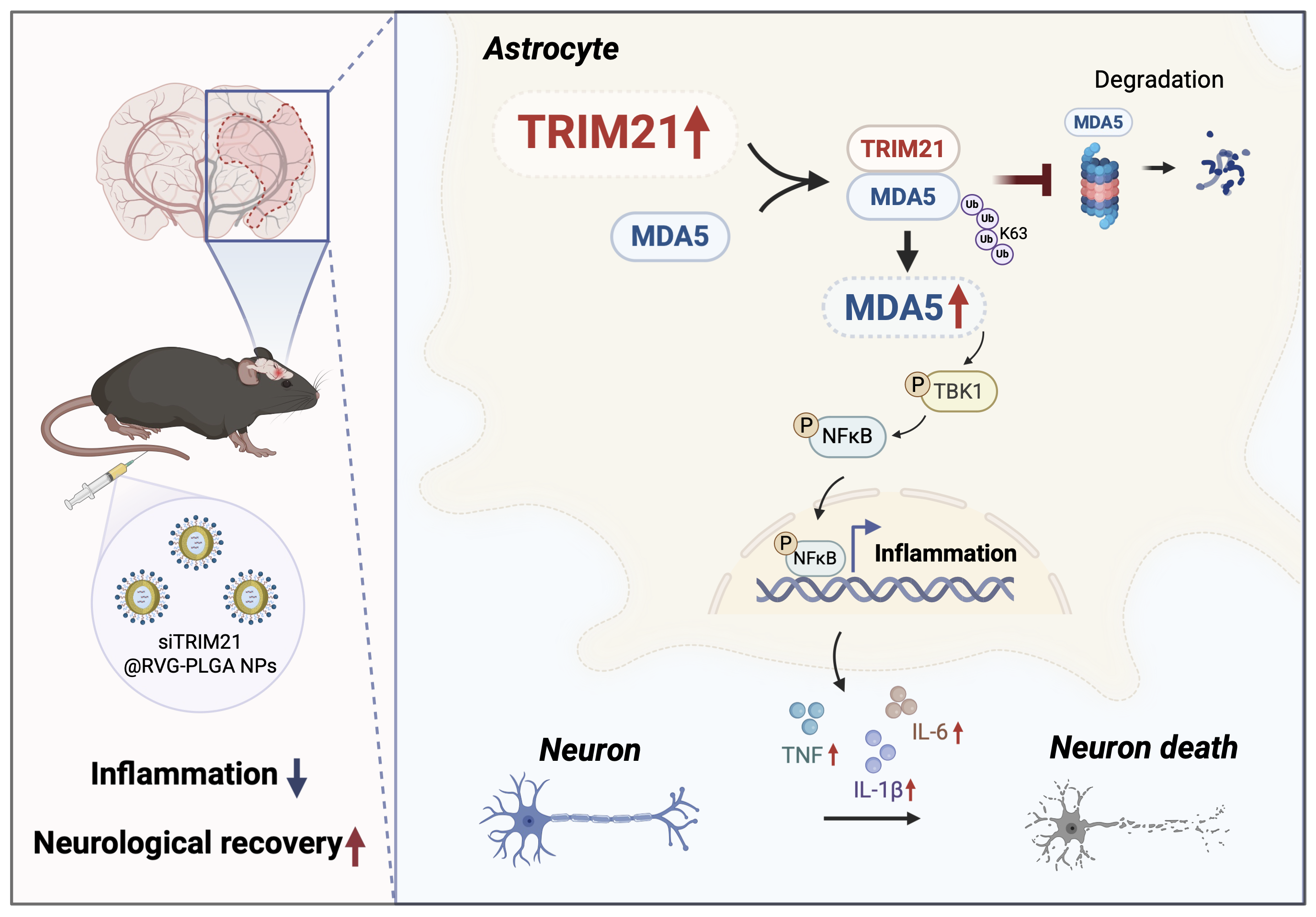

Supplement: Supplementary 1 — Graphical Abstract Figs. S1 to S7 Table S1 [file research.1200.f1.zip › Graphical abstract.tiff]
